# Prevalence and risk factors associated with undiagnosed diabetes in India: Insights from NFHS-5 national survey

**DOI:** 10.7189/jogh.13.04135

**Published:** 2023-12-08

**Authors:** Pravin Sahadevan, Vineet Kumar Kamal, Akhil Sasidharan, Bhavani Shankara Bagepally, Dolly Kumari, Anita Pal

**Affiliations:** 1 ICMR-National Institute of Epidemiology, Chennai, India; 2 Asian Development Research Institute, Patna, India; 3 Bihar Institute of Public Finance and Policy (BIPFP), Patna, India; 4 University of Hyderabad, Hyderabad, India

## Abstract

**Background:**

Undiagnosed diabetes is a significant public health concern in India, considering the accumulative burden of diabetes and its long-term complications. We have estimated the prevalence and factors associated with undiagnosed diabetes in India.

**Methods:**

We used data from the fifth round of the National Family Health Survey (NFHS-5, 2019-21) to estimate undiagnosed diabetes prevalence aged under 50 (15-49) years. A log-binomial model with survey-adjusted Poisson regression was used to estimate the prevalence risk ratio (PR) between undiagnosed diabetes and various factors. Multinomial logistic regression analysis was performed to examine the factors associated with diagnosed diabetes (vs. healthy) and undiagnosed diabetes (vs. healthy). All the analyses were survey-weighted and stratified by gender and reported with 95% confidence intervals.

**Results:**

The prevalence of diabetes for individuals aged 15-49 years was found to be 4.90% (4.80 to 5.00%) from the NFHS-5. Among them, the proportion of individuals with undiagnosed diabetes was 24.82% (24.07 to 25.59%), with higher among males (28.82% (26.45 to 31.30%)) than females (24.22% (23.44 to 25.01%)). The overall prevalence of undiagnosed diabetes was 1.22% (1.18 to 1.26%), with a higher prevalence among males (1.60% (1.46 to 1.76%)) than females (1.17% (1.13 to 1.21%)). Individuals who are middle-aged (45-49), have a higher body mass index (BMI), and are in a lower wealth index group, or live in the southern regions of India are at a higher risk of being undiagnosed for diabetes.

**Conclusion:**

One in every four having diabetes is undiagnosed. The study highlights the need for public health interventions to improve diabetes screening and access to health care, particularly among middle-aged individuals, and those with higher BMI, as well as addressing lifestyle and dietary factors. The findings also reveal disparities in diabetes burden among population subgroups in India, underscoring the need for targeted efforts.

Diabetes is a chronic metabolic disorder characterised by persistent hyperglycaemia, which can lead to complications including cardiovascular disease, kidney failure and blindness [1]. It is one of the fastest-growing health emergencies globally and a major public health concern in India, where the number of individuals with diabetes is expected to increase from 74.2 million in 2021 to 124.9 million by 2045 [2]. The Indian population is more prone to developing diabetes due to genetic susceptibility, increased abdominal obesity and higher prevalence of metabolic risk factors such as hypertension and dyslipidaemia [3,4]. Lifestyle factors such as changes in dietary habits, rapid urbanisation, and physical inactivity also add to the risk [5,6].

The International Diabetes Federation's (IDF) 2021 report shows the overall prevalence of diabetes among adults aged 20-79 in India at 8.3% (7.3 to 9.3%), slightly lower than the global prevalence of 10.5% [2]. The age-adjusted prevalence of diabetes in India is reported as 9.6% (8.5 to 10.6%) as per the IDF, 2021 [2]. The fifth round of the National Family Health Survey (NFHS-5), conducted in 2019-2021, reported a higher prevalence of high blood glucose level (>140 milligrammes per decilitre (mg/dl)) among men compared to women (15.6 vs. 13.5%) [7].

It is estimated that more than 50% of individuals with diabetes in India remain undiagnosed [7,8] or are unaware of their diabetes status [9,10]. IDF 2021 reports the prevalence of undiagnosed diabetes among the total diabetes population was 53.1%, totalling 39.4 million individuals [2]. Claypool et al. reported that 1.2% (95% confidence interval (CI) = 1.2 to 1.3%) of individuals aged 15-50 in India had undiagnosed diabetes, and 42% (95% CI = 40.7 to 43.4%) of those with high glucose levels were unaware of their diabetes status, based on the NFHS-4 survey data [11,12]. The average cost of diabetes-related expenses per person with diabetes in India in 2021 was US dollars (US$) 114.4 [2]. The current expenditure on diabetes in India is estimated at US$10.1 billion in 2021. It is projected to increase to US$15 billion by 2045, which can significantly burden the health care system [2]. This issue is further compounded by the high number of diabetes-related deaths, with India having 600 000 deaths in 2021, ranking it third in the world after China and the United States [2]. Considering the economic burden, diabetes alone exhausts 5 to 25% of the average Indian household's earnings [13]. These findings highlight the urgent need for increased efforts to identify and diagnose individuals at risk for diabetes to improve outcomes and reduce the overall burden of the disease in India.

This study investigates the prevalence and risk factors associated with undiagnosed diabetes among a nationally representative sample of men and women aged 15-49 years in India using NFHS-5 survey data. The study aims to provide insight into the characteristics of individuals with undiagnosed diabetes and the risk factors associated with undiagnosed diabetes in this population. The results of this study may aid future diabetes prevention and management efforts in India.

## METHODS

### Data source, study design and setting

A secondary data analysis was conducted using the survey data sets of the fifth round of the NFHS-5, conducted by the Indian Ministry of Health and Family Welfare (MoHFW) between 2019 and 2021. The NFHS-5 is a multi-round survey aimed at obtaining nationally representative data on population, health, and nutrition in India for each state and union territory as of 31 March 2017 [7]. The survey included participants' height, weight, blood pressure measurements, and random blood glucose levels using capillary blood glucose (CBG) with a glucose meter. Using a stratified two-stage sampling approach, a total of 724 115 women and 101 839 men were interviewed from 636 699 households, yielding a response rate of 97%. Appendix S1 in the **Online Supplementary Document** details NFHS-5′s methodology, covering sampling and data collection.

### Analytic sample

We analysed the data on 748 046 participants from the NFHS-5 survey of India. The sample population consisted of males and non-pregnant females, all under the age of 50 years, who were analysed separately (Figure S1 in the **Online Supplementary Document**).

### Study variables

The study assessed several socio-demographic and health-related variables, including age, wealth index, education, lifestyle habits, dietary habits, place of residence, body mass index (BMI), comorbid conditions and health care access. Body mass index was categorised according to the cut-off points proposed by the WHO and Asia-Pacific guidelines [14]. In this study, diagnosed diabetes was determined by participants' answering “yes” to any of the following questions: whether they currently have diabetes, whether they currently take a prescribed medication to lower blood glucose, or whether they were told by a doctor or other health professional that they have high blood glucose on two or more occasions. Participants with undiagnosed diabetes were identified by answering “no” or “don't know” to the diagnosed diabetes variable defined, accompanied by either opportunistic fasting glucose levels (≥126 mg/dl, with a fasting duration of ≥8 hours) or random glucose levels (≥200 mg/dl). The definition of diagnosed and undiagnosed diabetes was based on answers to questions and glucose levels, in accordance with established guidelines [15-17]. A complete overview of the study variables is provided in Appendix S2 in the **Online Supplementary Document**.

### Statistical analysis

We used descriptive statistics to assess the distribution of socio-demographic, behavioural, food-intake-related variables and comorbidities between diagnosed and undiagnosed in males, females and the overall population. We calculated the prevalence of undiagnosed diabetes and diagnosed diabetes with a 95% confidence interval (CI) by utilising sampling weights and survey-weighted proportions to account for the survey design, considering the sampling strategy and clustering effect. We used log-binomial models, adjusted for the survey design using survey-adjusted poisson regression, to evaluate the association of socio-demographic, behavioural, food intake-related variables and other potential comorbid conditions with undiagnosed diabetes. We quantified the association between factors and outcome variables using the prevalence risk ratio (PR) with its 95% CI. Additionally, we applied survey-adjusted univariate and multivariable multinomial logistic regression analysis to see the factors associated with diagnosed diabetes (vs. healthy) and undiagnosed diabetes (vs. healthy). We reported results using crude relative risk ratio (RRR) and adjusted RRR with its 95% CI. In all multivariable analyses, we included only those factors which turned out to be significant at 20% (or alpha = 0.20) in bivariable analysis. All analyses were conducted using survey-weighted methods stratified by gender. We considered all the *P*-values less than 0.05 as significant. We used Stata 17.0 for all statistical analyses [18].

### Patient and public involvement

No patients were involved in the development of the research question or the design of this study or the outcome measures. There is no plan to disseminate the results of the research to study participants.

## RESULTS

### General characteristics of the population

The NFHS-5 (2019-2021) survey included a total of 788 974 individuals, with both males and non-pregnant females under the age of 50 (15-49) years. We removed 40 928 individuals' data that were missing time and/or glucose values for “no” or “don’t know” group in self-reported diabetes. This yielded a sample size of 748 046, as shown in Figure S1 in the **Online Supplementary Document**. Eighty-eight percent of the surveyed individuals were women. The general characteristics of the surveyed variables among males and females, and the overall total sampled data, are described in Table S1 in the **Online Supplementary Document**. Sixty-two percent of the surveyed individuals were between 15 years and 35 years of age. In the surveyed population, one-fourth (24.82%) of individuals with diabetes were undiagnosed. Of those diagnosed, one-third (36.38%) were undergoing treatment; among those being treated, approximately two-thirds (64.09%) had adequate glycaemic control.

### Socio-demographic and behavioural profile of undiagnosed diabetes population

**Table 1** displays the distribution of socio-demographic, behavioural, food-intake-related variables and comorbidities conditions between diagnosed and undiagnosed diabetes groups stratified by gender and overall. Individuals with undiagnosed diabetes were found to be more in rural regions (70%), with the southern region of India having the highest rates of diabetes and undiagnosed diabetes. Undiagnosed diabetes was found to be higher among individuals without any comorbidities (88%). Furthermore, nearly half of the individuals with undiagnosed diabetes cases were from the higher wealth index or had access to health care.

**Table 1:** General characteristics of the diabetes population

Characteristics	Males			Females			Overall	
	**Diagnosed diabetes, n (%)**	**Undiagnosed diabetes, n (%)**	**Total diabetes, n (%)**	**Diagnosed diabetes, n (%)**	**Undiagnosed diabetes, n (%)**	**Total diabetes, n (%)**	**Undiagnosed diabetes, n (%)**	**Total diabetes, n (%)**
**Age in years, mean (SD)**	36.56 (9.50)	36.81 (8.57)	36.63 (9.24)	36.79 (9.37)	37.39 (8.71)	36.93 (9.22)	37.30 (8.69)	36.89 (9.22)
**Age categories**								
15-24	425 (14.16)	138 (11.28)	563 (13.33)	2922 (13.30)	671 (10.10)	3593 (12.35)	809 (10.28)	4156 (12.65)
25-34	649 (21.63)	272 (22.24)	921 (21.80)	4910 (22.34)	1479 (22.25)	6389 (22.32)	1751 (22.25)	7310 (22.26)
35-44	1108 (36.92)	526 (43.01)	1634 (38.68)	8050 (36.63)	2703 (40.67)	10 753 (37.57)	3229 (41.03)	12 387 (37.71)
45-49	819 (27.29)	287 (23.47)	1106 (26.18)	6094 (27.73)	1793 (26.98)	7887 (27.56)	2080 (26.63)	8993 (27.38)
**Wealth index**								
Poorest	362 (12.06)	173 (14.15)	535 (12.67)	2925 (13.31)	1115 (16.78)	4040 (14.12)	1288 (16.37)	4575 (13.93)
Poorer	539 (17.96)	261 (21.34)	800 (18.94)	3922 (17.85)	1309 (19.70)	5231 (18.28)	1570 (19.95)	6031 (18.36)
Middle	662 (22.06)	280 (22.89)	942 (22.30)	4574 (20.81)	1501 (22.59)	6075 (21.22)	1781 (22.63)	7017 (21.36)
Richer	735 (24.49)	286 (23.39)	1021 (24.17)	5283 (24.04)	1522 (22.90)	6805 (23.78)	1808 (22.98)	7826 (23.83)
Richest	703 (23.43)	223 (18.23)	926 (21.92)	5272 (23.99)	1199 (18.04)	6471 (22.61)	1422 (18.07)	7397 (22.52)
**Education**								
No education	311 (10.36)	120 (9.81)	431 (10.20)	5485 (24.96)	2165 (32.58)	7650 (26.73)	2285 (29.04)	8081 (24.60)
Primary	319 (10.63)	164 (13.41)	483 (11.43)	3037 (13.82)	984 (14.81)	4021 (14.05)	1148 (14.59)	4504 (13.71)
Secondary	1738 (57.91)	729 (59.61)	2467 (58.40)	10 553 (48.02)	2912 (43.82)	13 465 (47.04)	3641 (46.27)	15 932 (48.51)
Higher	633 (21.09)	210 (17.17)	843 (19.96)	2901 (13.20)	585 (8.80)	3486 (12.18)	795 (10.10)	4329 (13.18)
**Place of residence**								
Urban	974 (32.46)	380 (31.07)	1354 (32.05)	7326 (33.34)	1945 (29.27)	9271 (32.39)	2325 (29.55)	10 625 (32.35)
Rural	2027 (67.54)	843 (68.93)	2870 (67.95)	14 650 (66.66)	4701 (70.73)	19 351 (67.61)	5544 (70.45)	22 221 (67.65)
**Region of country**								
North	578 (19.26)	160 (13.08)	738 (17.47)	4635 (21.09)	854 (12.85)	5489 (19.18)	1014 (12.89)	6227 (18.96)
Central	525 (17.49)	235 (19.22)	760 (17.99)	3991 (18.16)	1226 (18.45)	5217 (18.23)	1461 (18.57)	5977 (18.20)
East	519 (17.29)	179 (14.64)	698 (16.52)	3446 (15.68)	1117 (16.81)	4563 (15.94)	1296 (16.47)	5261 (16.02)
Northeast	407 (13.56)	159 (13.00)	566 (13.40)	3228 (14.69)	708 (10.65)	3963 (13.75)	867 (11.02)	4502 (13.71)
West	278 (9.26)	194 (15.86)	472 (11.17)	1699 (7.73)	891 (13.41)	2590 (9.05)	1085 (13.79)	3062 (9.32)
South	694 (23.13)	296 (24.20)	990 (23.44)	4977 (22.65)	1850 (27.84)	6827 (23.85)	2146 (27.27)	7817 (23.80)
**Milk or curd use**								
Never/occasionally	676 (22.53)	278 (22.73)	954 (22.59)	5611 (25.53)	1891 (28.45)	7502 (26.21)	2169 (27.56)	8456 (25.74)
Daily	1511 (50.35)	602 (49.22)	2113 (50.02)	11 366 (51.72)	3321 (49.97)	14 687 (51.31)	3923 (49.85)	16 800 (51.15)
Weekly	814 (27.12)	343 (28.05)	1157 (27.39)	4999 (22.75)	1434 (21.58)	6433 (22.48)	1777 (22.58)	7590 (23.11)
**Pulses or beans use**								
Never/occasionally	227 (7.56)	95 (7.77)	322 (7.62)	2034 (9.26)	539 (8.11)	2573 (8.99)	634 (8.06)	2875 (8.81)
Daily	1546 (51.52)	583 (47.67)	2129 (50.40)	10 885 (49.53)	3127 (47.05)	14 012 (48.96)	3710 (47.15)	16 141 (49.14)
Weekly	1228 (40.92)	545 (44.56)	1773 (41.97)	9057 (41.21)	2980 (44.84)	12 037 (42.06)	3525 (44.80)	13 810 (42.04)
**Green leafy vegetables**								
Never/occasionally	222 (7.40)	90 (7.36)	312 (7.39)	2002 (9.11)	698 (10.50)	2700 (9.43)	788 (10.01)	3012 (9.17)
Daily	1585 (52.82)	623 (50.94)	2208 (52.27)	11 748 (53.46)	3261 (49.07)	15 009 (52.44)	3884 (49.36)	17 217 (52.42)
Weekly	1194 (39.79)	510 (41.70)	1704 (40.34)	8226 (37.43)	2687 (40.43)	10 913 (38.13)	3197 (40.63)	12 617 (38.41)
**Fruits**								
Never/occasionally	1195 (39.82)	553 (45.22)	1748 (41.38)	9768 (44.45)	3357 (50.51)	13 125 (45.86)	3910 (49.69)	14 873 (45.28)
Daily	492 (16.39)	149 (12.18)	641 (15.18)	3455 (15.72)	757 (11.39)	4212 (14.72)	906 (11.51)	4853 (14.78)
Weekly	1314 (43.79)	521 (42.60)	1835 (43.44)	8753 (39.83)	2532 (38.10)	11 285 (39.43)	3053 (38.80)	13 120 (39.94)
**Eggs**								
Never/occasionally	1355 (45.15)	575 (47.02)	1930 (45.69)	11 614 (52.85)	3477 (52.32)	15 091 (52.73)	4052 (51.49)	17 021 (51.82)
Daily	266 (8.86)	72 (5.89)	338 (8.00)	1470 (6.69)	348 (5.24)	1818 (6.35)	420(5.34)	2156 (6.56)
Weekly	1380 (45.98)	576 (47.02)	1956 (45.69)	8892 (40.46)	2821 (42.45)	11 713 (40.92)	3397 (43.17)	13 669 (41.62)
**Fish**								
Never/occasionally	1726 (57.51)	720 (58.87)	2446 (57.91)	13 420 (61.07)	4268 (64.22)	17 688 (61.80)	4988 (63.39)	20 134 (6.12)
Daily	164 (5.46)	59 (4.82)	223 (5.28)	1486 (6.76)	301 (4.53)	1787 (6.24)	360 (4.57)	2010 (6.12)
Weekly	1111 (37.02)	444 (36.30)	1555 (36.81)	7070 (32.17)	2077 (31.25)	9147 (31.96)	2521 (32.04)	10 702 (32.58)
**Chicken or meat**								
Never/occasionally	1666 (55.51)	684 (55.93)	2350 (53.63)	13 634 (62.04)	4034 (60.70)	17 668 (61.73)	4718 (59.96)	20 018 (60.95)
Daily	92 (3.07)	23 (1.88)	115 (2.72)	438 (1.99)	110 (1.66)	548 (1.91)	133 (1.69)	663 (2.02)
Weekly	1243 (41.42)	516 (42.19)	1759 (41.64)	7904 (35.97)	2502 (37.65)	10 406 (36.36)	3018 (38.35)	12 165 (37.04)
**Fried food**								
Never/occasionally	1645 (54.82)	706 (57.73)	2351 (55.66)	12 184 (55.44)	4004 (60.25)	16 188 (56.56)	4710 (59.86)	18 539 (56.44)
Daily	331 (11.03)	126 (10.30)	457 (10.82)	2417 (11.00)	500 (7.52)	2917 (10.19)	626 (7.96)	3374 (10.27)
Weekly	1025 (34.16)	391 (31.97)	1416 (33.52)	7375 (33.56)	2142 (32.23)	9517 (33.25)	2533 (32.19)	10 933 (33.29)
**Aerated drinks**								
Never/occasionally	2236 (74.51)	959 (78.41)	3195 (75.64)	18 021 (82.00)	5664 (85.22)	23 685 (82.75)	6623 (84.17)	26 880 (81.84)
Daily	160 (5.33)	43 (3.52)	203 (4.81)	743 (3.38)	171 (2.57)	914 (3.19)	214 (2.72)	1117 (3.40)
Weekly	605 (20.16)	221 (18.07)	826 (19.55)	3212 (14.62)	811 (12.20)	4023 (14.06)	1032 (13.11)	4849 (14.76)
**Tobacco usage**								
No	1978 (65.91)	776 (63.45)	2754 (65.20)	21 153 (96.26)	6351 (95.56)	27 504 (96.09)	7127 (90.57)	30 258 (92.12)
Yes	1023 (34.09)	447 (36.55)	1470 (34.80)	823 (3.74)	295 (4.44)	1118 (3.91)	742 (9.43)	2588 (7.88)
**Alcohol usage**								
No	2075 (69.14)	810 (66.23)	2885 (68.30)	21 560 (98.11)	6532 (98.28)	28 092 (98.15)	7342 (93.30)	30 977 (94.31)
Yes	926 (30.86)	413 (33.77)	1339 (31.70)	416 (1.89)	114 (1.72)	530 (1.85)	527 (6.70)	1869 (5.69)
**Body mass index**								
Underweight (<18.5)	232 (7.92)	102 (8.38)	334 (8.05)	1921 (8.88)	612 (9.23)	2533 (8.97)	714 (9.10)	2867 (8.85)
Normal (18.50-22.99)	985 (33.61)	366 (30.07)	1351 (32.57)	7223 (33.40)	1982 (29.90)	9205 (32.58)	2348 (29.93)	10 556 (32.58)
Overweight (23-24.99)	606 (20.68)	237 (19.47)	843 (20.32)	3580 (16.56)	1028 (15.51)	4608 (16.31)	1265 (16.12)	5451 (16.82)
Obese1 (25-29.99)	859 (29.31)	372 (30.57)	1231 (29.68)	5873 (27.16)	1930 (29.12)	7803 (37.62)	2302 (29.34)	9034 (27.88)
Obese2 (≥30)	249 (8.50)	140 (11.50)	389 (9.38)	3027 (14.00)	1076 (16.23)	4103 (14.52)	1216 (15.50)	4492 (13.86)
**Comorbidities**								
None	2432 (81.04)	1142 (93.38)	3574 (84.61)	15 324 (69.73)	5764 (86.73)	21 088 (73.68)	6906 (87.76)	24662 (75.08)
One	426 (14.20)	68 (5.56)	494 (11.40)	4993 (22.72)	763 (11.48)	5756 (20.11)	831 (10.56)	6250 (19.03)
More than one	143 (4.77)	13 (1.06)	156 (3.69)	1659 (7.55)	119 (1.79)	1778 (6.21)	132 (1.68)	1934 (5.89)
**Access to health care**								
No	1152 (38.39)	528 (43.17)	1680 (39.77)	10 949 (49.82)	3377 (50.81)	14 326 (50.05)	3905 (49.63)	16 006 (48.73)
Yes	1849 (61.61)	695 (56.83)	2544 (60.23)	11 027 (50.18)	3269 (49.19)	14 296 (49.95)	3964 (50.37)	16 840 (51.27)

SD – standard deviation

### Survey-adjusted prevalence of diabetes (undiagnosed and diagnosed)

**Figure 1:**
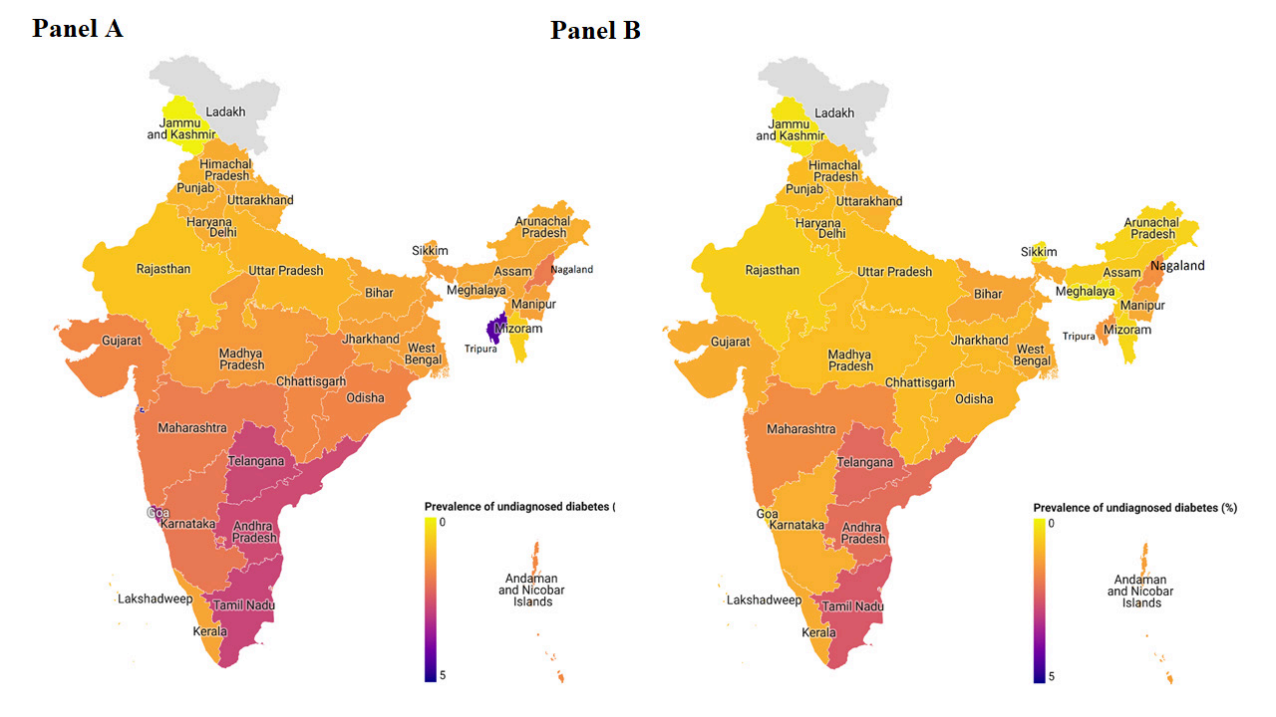
Prevalence of undiagnosed diabetes in Indian States. **Panel A** and **Panel B** represent the prevalence of undiagnosed diabetes in different Indian states among males and females, respectively. The color intensity varies from 0 (lowest prevalence) to 1 (highest prevalence).

The prevalence of diabetes for women and men aged 15-49 years was 4.90% (4.80 to 5.00%), with a higher prevalence in males at 5.56% (5.25 to 5.89%) and females at 4.81% (4.72 to 4.90%). The proportion of individuals with undiagnosed diabetes was 24.82% (24.07 to 25.59%) and is higher in males (28.82% (26.45 to 31.30%)) than in females (24.22% (23.44 to 25.01%)). The overall prevalence of undiagnosed diabetes was 1.22% (1.18 to 1.26%) and is higher among males (1.60% (1.46 to 1.76%)) than in females (1.17% (1.13 to 1.21%)). The overall prevalence of diagnosed diabetes was 3.68% (3.60 to 3.77%) and is higher in males (3.96% (3.69 to 4.25%)) than in females (3.65% (3.56 to 3.74%)). Table S2 in the **Online Supplementary Document**, provides data on the prevalence of undiagnosed diabetes among different Indian states, and Table S3 in the **Online Supplementary Document**, provides the prevalence of undiagnosed diabetes among individuals with health care access by state. The prevalence of undiagnosed diabetes in the male and female populations of Indian states, respectively (**Figure 1**, panels A and B). The prevalence of diabetes and undiagnosed diabetes is given in **Table 2**.

**Table 2:** Prevalence of diabetes and undiagnosed diabetes in India

Diabetes diagnostic criteria*	Current study†	NFHS 5‡
	Opportunistic fasting (≥8 hours) glucose level ≥126 mg/dl ‘OR’ a random glucose level ≥200 mg/dl	>140 mg/dl
**Prevalence of diabetes**		
Men	5.56 (5.25-5.89)	12.76 (12.29-13.24)
Women	4.81 (4.72-4.90)	10.00 (9.88-10.14)
Overall	4.90 (4.80-5.00)	10.32 (10.20-10.45)
**Prevalence of undiagnosed diabetes**		
Men	1.60 (1.46-1.76)	8.80 (8.42-9.20)
Women	1.17 (1.13-1.21)	6.36 (6.27-6.46)
Overall	1.22 (1.18-1.26)	6.64 (6.54-6.74)

NFHS – National Family Health Survey, mg – milligramme, dl – decilitre

*Along with diagnosed diabetes.

†As per Indian ICMR guidelines for management of Type 2 Diabetes 2018 and other standard guidelines.

‡As per NFHS case definition for high blood glucose.

**Figure 2:**
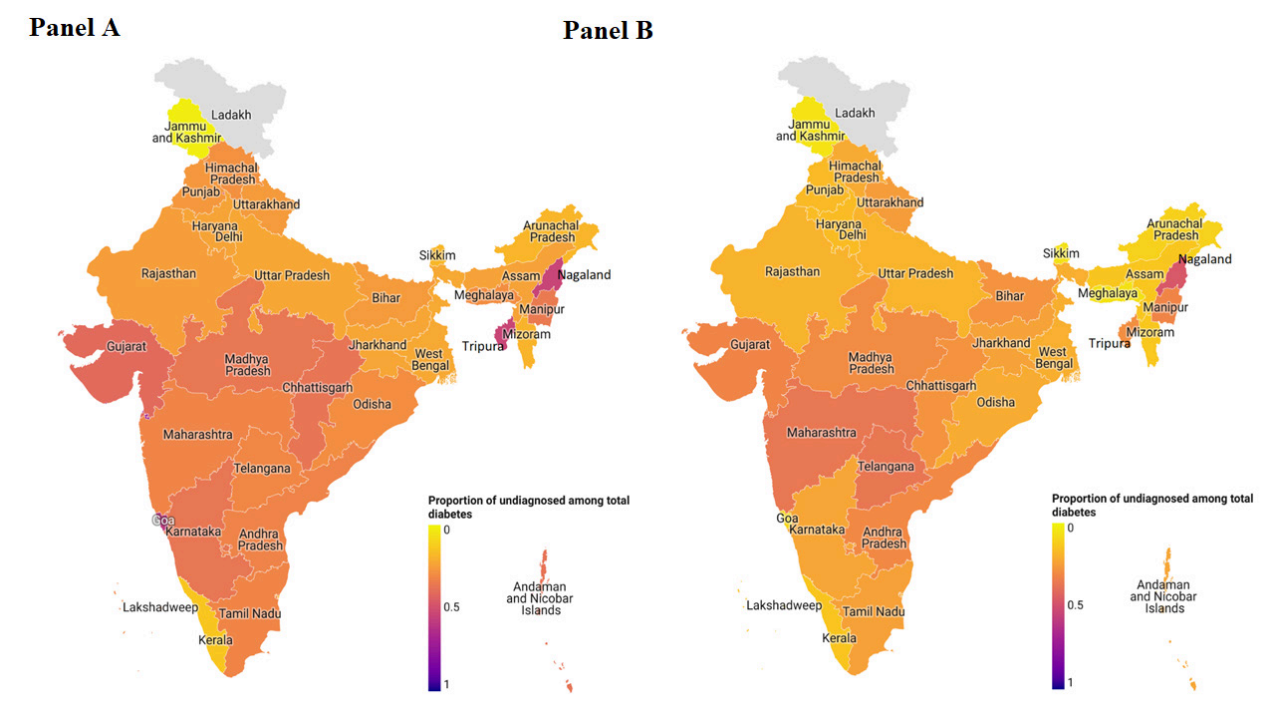
Proportion of undiagnosed among total diabetes by Indian States. **Panel A** and **Panel B** represent the proportion of undiagnosed diabetes among the total diabetes cases in Indian states for males and females, respectively. The color intensity ranges from 0 (lowest proportion) to 1 (highest proportion).

The bivariable association of socio-demographic, behavioural, and other lifestyle factors with total diabetes outcome (undiagnosed and diagnosed diabetes) stratified by gender is presented in Table S4 in the **Online Supplementary Document**. The proportion of individuals with undiagnosed diabetes among the total number of diabetes in the male and female populations of Indian states is presented in **Figure 2**, panels A and B, and Table S5 in the **Online Supplementary Document**.

Prevalence risk ratios of undiagnosed diabetes for men and women across socio-demographic, biological, and lifestyle factors

**Table 3** shows the results of Log-binomial models using survey-adjusted Poisson regression in the form of unadjusted and adjusted prevalence ratio of undiagnosed diabetes vs. diagnosed diabetes in males and females. Factors including age, wealth, education, region of residence, dietary habits, access to health care and comorbidities have a significant impact on the unadjusted prevalence RR of undiagnosed diabetes vs. diagnosed diabetes in both males and females.

**Table 3:** Prevalence ratio of undiagnosed vs. diagnosed diabetes in males and females

Characteristics	Undiagnosed vs. diagnosed diabetes prevalence ratio (95% CI)		Undiagnosed vs. diagnosed diabetes prevalence ratio (95% CI)	
	**Male**		**Female**	
	**Unadjusted prevalence ratio (95% CI)**	**Adjusted prevalence ratio (95% CI)**	**Unadjusted prevalence ratio (95% CI)**	**Adjusted prevalence ratio (95% CI)**
**Age group (ref: 15-24)**				
25-34	1.21 (0.91-1.62)	1.20 (0.91-1.58)	1.16 (1.04-1.29)*	1.12 (0.99-1.26)
35-44	1.19 (0.91-1.56)	1.21 (0.92-1.58)	1.25 (1.13-1.39)*	1.20 (1.07-1.35)*
45-49	1.01 (0.76-1.34)	1.05 (0.78-1.41)	1.05 (0.94-1.18)	1.05 (0.92-1.19)
**Wealth index (ref: richest)**				
Poorest	1.35 (0.99-1.86)	1.55 (1.14-2.11)*	1.45 (1.30-1.61)*	1.33 (1.17-1.52)*
Poor	1.49 (1.14-1.94)*	1.58 (1.24-2.02)*	1.31 (1.18-1.45)*	1.19 (1.06-1.33)*
Middle	1.46 (1.12-1.90)*	1.50 (1.19-1.89)*	1.31 (1.18-1.45)*	1.18 (1.06-1.31)*
Richer	1.31 (1.00-1.71)*	1.28 (1.01-1.63)*	1.22 (1.10-1.34)*	1.12 (1.01-1.23)*
**Educational level (ref: higher)**				
No education	0.99 (0.70-1.39)		1.55 (1.35-1.77)*	1.33 (1.16-1.53)*
Primary	1.13 (0.83-1.54)		1.38 (1.19-1.59)*	1.22 (1.05-1.40)*
Secondary	1.07 (0.85-1.34)		1.25 (1.10-1.43)*	1.14 (1.01-1.29)*
**Place of residence (ref: rural)**				
Urban	0.90 (0.74-1.08)		0.84 (0.78-0.89)*	0.89 (0.82-0.97)*
**Region of country (ref: north)**				
Central	1.31 (1.06-1.63)*	1.15 (0.92-1.43)	1.23 (1.11-1.36)*	1.10(0.98,1.22)
East	1.14 (0.87-1.50)	0.96 (0.72-1.27)	1.31 (1.17-1.46)*	1.30(1.15,1.46)*
Northeast	1.39 (1.02-1.89)*	1.11 (0.81-1.52)	0.92 (0.80-1.05)	0.93(0.79,1.09)
West	1.65 (1.31-2.10)*	1.52 (1.21-1.90)*	1.92 (1.71-2.16)*	1.82(1.63,2.03)*
South	1.43 (1.14-1.78)*	1.30 (1.03-1.63)*	1.41 (1.28-1.55)*	1.34(1.20,1.49)*
**Milk/curd (ref: daily)**				
Weekly	0.99 (0.78-1.26)		0.99 (0.92-1.08)	0.99(0.92,1.07)
Never/occasionally	0.98 (0.80-1.22)		1.10 (1.02-1.19)*	1.10(1.01,1.19)*
**Pulses/beans (ref: daily)**				
Weekly	0.96 (0.81-1.14)		1.14 (1.07-1.22)*	1.06(0.99,1.13)
Never/occasionally	0.88 (0.65-1.19)		0.96 (0.86-1.08)	0.87(0.77,0.98)*
**Green leafy vegetables (ref: daily)**				
Weekly	1.00 (0.84-1.19)		1.14 (1.07-1.22)*	1.06(0.99,1.13)
Never/occasionally	1.24 (0.87-1.78)		1.19 (1.08-1.32)*	1.15(1.03,1.27)*
**Fruits (ref: daily)**				
Weekly	1.18 (0.87,1.61)		1.21 (1.09-1.34)*	1.04(0.94,1.15)
Never/occasionally	1.22 (0.90,1.65)		1.29 (1.17-1.42)*	1.02(0.92,1.13)
**Eggs (ref: daily)**				
Weekly	1.67 (1.18-2.37)*	1.43 (1.02-2.02)*	1.19 (1.03-1.38)*	1.09(0.95,1.25)
Never/occasionally	1.54 (1.07-2.22)*	1.29 (0.89-1.86)	1.11 (0.96-1.29)	0.99(0.85,1.14)
**Fish (ref: daily)**				
Weekly	0.93 (0.65-1.33)		1.31 (1.11-1.56)*	1.14(0.97,1.34)
Never/occasionally	0.97 (0.68-1.38)		1.37 (1.16-1.61)*	1.27(1.08,1.50)*
**Chicken (ref: daily)**				
Weekly	1.52 (0.79-2.96)		0.99 (0.78-1.26)	
Never/occasionally	1.43 (0.74-2.77)		0.91 (0.72-1.16)	
**Fried food (ref: daily)**				
Weekly	0.93 (0.67-1.28)		1.32 (1.16-1.50)*	1.15(1.01,1.31)*
Never/occasionally	0.96 (0.72-1.28)		1.43 (1.26-1.63)*	1.20(1.05,1.36)*
**Aerated drinks (ref: daily)**				
Weekly	1.32 (0.85-2.06)	1.16 (0.77-1.75)	0.95 (0.76-1.20)	
Never/occasionally	1.32 (0.88-1.98)	1.10 (0.75-1.62)	1.14 (0.92-1.42)	
**Tobacco usage (ref: no)**				
Yes	1.07 (0.87-1.30)		1.30 (1.12-1.50)*	1.22(1.06,1.41)*
**Body mass index (ref: normal)**				
Underweight	0.93 (0.69-1.25)	0.90 (0.67-1.22)	1.04 (0.94-1.16)	0.98(0.88,1.09)
Overweight	1.06 (0.84-1.34)	1.10 (0.88-1.37)	1.05 (0.95-1.15)	1.10(1.00,1.21)*
Obese 1	1.04 (0.82-1.32)	1.10 (0.88-1.38)	1.14 (1.05-1.23)*	1.25(1.16,1.35)*
Obese 2	1.50 (1.12-2.00)*	1.72 (1.33-2.22)*	1.13 (1.04-1.24)*	1.40(1.27,1.53)*
**Number of comorbidities (ref: more than one)**				
No	3.55 (1.66-7.57)*	3.25 (1.53-6.87)*	4.14 (3.29-5.21)*	4.17(3.31,5.24)*
One	1.35 (0.56-3.22)	1.23 (0.52-2.94)	2.00 (1.57-2.55)*	2.00(1.58,2.56)*
**Healthcare access (ref: yes)**				
No	1.16 (0.99-1.36)	1.18 (1.01-1.38)*	1.08 (1.01-1.14)*	1.03(0.97,1.09)
**Alcohol usage (ref: no)**				
Yes	1.05 (0.86-1.29)		1.11 (0.88-1.40)	

CI – confidence interval

*Significant at *P* <0.05.

Adjusted prevalence risk ratios of undiagnosed diabetes for men and women across socio-demographic, biological, and lifestyle factors

The crude prevalence risk ratio was adjusted for significant variables at a 20% significance level in bivariable analysis to control for factors that may bias the results. The results are presented as adjusted prevalence ratios ((aPR), 95% CI) for undiagnosed diabetes vs. diagnosed diabetes in **Table 3**.

#### Females

Urban females had a 11% lower prevalence of undiagnosed diabetes than rural females (aPR = 0.89; 95% CI = 0.82 to 0.97). Compared to older age group 45-49, females aged 35-44 had a 20% higher prevalence of undiagnosed diabetes (aPR = 1.20; 95% CI = 1.07 to 1.35). Females in the lowest wealth index group had a 33% higher prevalence of undiagnosed diabetes than those in the highest wealth index group (aPR = 1.33; 95% CI = 1.17 to 1.52). Similarly, females with no education had a 33% higher prevalence of undiagnosed diabetes than those with higher education (aPR = 1.33; 95% CI = 1.16 to 1.53). Females living in the geographic west of India had an 82% higher prevalence of undiagnosed diabetes than those living in the geographic north (aPR = 1.82; 95% CI = 1.63 to 2.03).

Among modifiable risk factors, females who consumed green leafy vegetables never or occasionally had a 15% higher prevalence of undiagnosed diabetes than those who consumed them daily (aPR = 1.15; 95% CI = 1.03 to 1.27). Females who consumed fish never or occasionally had a 27% higher prevalence of undiagnosed diabetes than those who consumed them daily (aPR = 1.27; 95% CI = 1.08 to 1.50). Females who consumed milk/curd never or occasionally had a 10% higher prevalence of undiagnosed diabetes than those who consumed them daily (aPR = 1.10; 95% CI = 1.01 to 1.19). Females who consumed fried food never or occasionally had a 20% higher prevalence of undiagnosed diabetes than those who consumed them daily (aPR = 1.20; 95% CI = 1.01 to 1.19). Conversely, females who consumed pulses/beans never or occasionally had a 13% lower prevalence of undiagnosed diabetes than those who consumed them daily (aPR = 0.87; 95% CI = 0.77 to 0.98). Females who use tobacco had a 22% higher prevalence of undiagnosed diabetes (aPR = 1.22; 95% CI = 1.06 to 1.41). Females in the “obese 2” BMI category had a 40% higher prevalence of undiagnosed diabetes than with a normal BMI (aPR = 1.40; 95% CI = 1.27 to 1.53). Females who had no comorbidities had a higher prevalence of undiagnosed diabetes than those with more than one comorbidity (aPR = 4.17; 95% CI = 3.31 to 5.24).

#### Males

Males in the lowest wealth index group had a 55% higher prevalence of undiagnosed diabetes than those in the highest wealth index group (aPR = 1.55; 95% CI = 1.14 to 2.11). Males living in the geographic west of India had a 52% higher prevalence of undiagnosed diabetes than those living in the geographic north (aPR = 1.52; 95% CI = 1.21 to 1.90). Among modifiable risk factors, males who consumed eggs weekly had a 43% higher prevalence of undiagnosed diabetes than those who consumed them daily (aPR = 1.43; 95% CI = 1.02 to 2.02). Males in the “obese 2” category had a 72% higher prevalence of undiagnosed diabetes (aPR = 1.72; 95% CI = 1.33 to 2.22). Males who had no comorbidities had almost three times higher prevalence of undiagnosed diabetes than those with more than one comorbidity (aPR = 3.25; 95% CI = 1.53 to 6.87). Males without health care access had a 18% higher prevalence of undiagnosed diabetes (aPR = 1.18; 95% CI = 1.01 to 1.38).

### Factors associated with diagnosed diabetes (vs. healthy) and undiagnosed diabetes (vs. healthy): Results from multinomial logistic regression analysis

Table S6 in the **Online Supplementary Document** shows the association of socio-demographic, behavioural, food-intake-related variables and comorbidities conditions with diabetes using the χ^2^ test. The crude and adjusted associations of different factors with diagnosed diabetes (vs. healthy) and undiagnosed diabetes (vs. healthy) using univariate and multivariable multinomial logistic regression analyses, respectively in both males and females separately are presented as RRR) in **Table 4**. The crude findings indicate that residing in urban areas, increasing age, higher wealth, and higher BMI categories were associated with increased risks of both diagnosed and undiagnosed diabetes in males and females. In male participants, access to health care and the absence of comorbidities are associated with reduced risks, while in female participants, lower educational levels and daily consumption of specific foods increase the risks. Based on the adjusted RRR, the study’s findings suggest that certain demographic, dietary and modifiable risk factors are associated with an increased risk of undiagnosed diabetes and diagnosed diabetes in females and males, and are as follows:

**Table 4:** Relative risk ratio for undiagnosed diabetes vs. healthy and diagnosed diabetes vs. healthy in males and females

Characteristics	Male				Female			
	**Undiagnosed diabetes vs. healthy**		**Diagnosed diabetes vs. healthy**		**Undiagnosed diabetes vs. healthy**		**Diagnosed diabetes vs. healthy**	
	**Crude RRR (95% CI)**	**Adjusted RRR (95% CI)**	**Crude RRR (95% CI)**	**Adjusted RRR (95% CI)**	**Crude RRR (95% CI)**	**Adjusted RRR (95% CI)**	**Crude RRR (95% CI)**	**Adjusted RRR (95% CI)**
**Age group (ref: 15-24)**								
25-34 years	2.17 (1.52-3.10)*	1.67 (1.18-2.36)*	1.66 (1.35-2.03)*	1.32 (1.07-1.63)*	2.37 (2.10-2.67)*	1.77 (1.56-2.00)*	1.96 (1.83-2.11)	1.53 (1.41-1.65)*
35-44 years	4.45 (3.28-6.03)*	3.20 (2.35-4.36)*	3.50 (2.82-4.34)*	2.55 (2.05-3.18)*	5.58 (4.98-6.24)*	3.54 (3.13-4.00)*	4.17 (3.90-4.45)	2.77 (2.57-2.98)*
45-49 years	7.24 (5.25-9.97)*	5.24 (3.77-7.30)*	7.16 (5.74-8.94)*	4.99 (3.96-6.28)*	7.75 (6.84-8.79)*	4.75 (4.11-5.50)*	7.27 (6.77-7.80)	4.50 (4.15-4.87)*
**Wealth index (ref: poorest)**								
Poorer	1.44 (1.05-1.97)*	1.32 (0.95-1.83)	1.25 (0.96-1.63)	1.29 (0.97-1.73)	1.11 (0.99-1.25)	0.98 (0.87-1.09)	1.28 (1.19-1.37)*	1.12 (1.04-1.21)*
Middle	1.71 (1.25-2.33)*	1.36 (0.96-1.92)	1.53 (1.21-1.95)*	1.53 (1.18-1.99)*	1.36 (1.22-1.51)*	1.00 (0.89-1.13)	1.56 (1.45-1.68)*	1.15 (1.06-1.24)*
Richer	1.87 (1.34-2.61)*	1.28 (0.88-1.85)	1.96 (1.55-2.48)*	1.96 (1.47-2.59)*	1.54 (1.38-1.72)*	1.06 (0.93-1.21)	1.95 (1.81-2.10)*	1.27 (1.17-1.38)*
Richest	1.60 (1.12-2.30)*	1.03 (0.69-1.55)	2.42 (1.86-3.15)*	2.27 (1.67-3.09)*	1.42 (1.26-1.60)*	0.99 (0.85-1.17)	2.31 (2.14-2.48)*	1.32 (1.20-1.46)*
**Education level (ref: higher)**								
No education	0.96 (0.65-1.42)		0.97 (0.75-1.26)		2.04 (1.75-2.38)*	1.39 (1.16-1.67)*	1.16 (1.10-1.24)*	0.94 (0.86-1.02)
Primary	1.09 (0.76-1.56)		0.91 (0.71-1.17)		1.94 (1.64-2.29)*	1.38 (1.15-1.65)*	1.29 (1.19-1.39)*	1.05 (0.97-1.15)
Secondary	1.02 (0.78-1.35)		0.93 (0.78-1.11)		1.35 (1.17-1.57)*	1.30 (1.11-1.52)*	1.02 (0.96-1.08)	1.08 (1.01-1.15)*
**Place of residence (ref: rural)**								
Urban	1.15 (0.92-1.43)	0.96 (0.76-1.23)	1.33 (1.13-1.57)*	0.95 (0.80-1.13)	1.23 (1.14-1.33)*	0.97 (0.87-1.07)	1.57 (1.49-1.66)*	1.12 (1.05-1.19)*
**Region of country (ref: North)**								
Central	1.34 (1.04-1.72)*	1.50 (1.14-1.99)*	0.93 (0.78-1.11)	1.29 (1.07-1.56)*	1.16 (1.03-1.29)*	1.31 (1.16-1.48)*	0.90 (0.83-0.96)*	1.19 (1.10-1.28)*
East	1.52 (1.13-2.05)*	1.79 (1.25-2.57)*	1.28 (1.05-1.56)*	1.97 (1.56-2.49)*	1.59 (1.41-1.80)*	1.80 (1.56-2.07)*	1.14 (1.06-1.22)*	1.29 (1.18-1.41)*
Northeast	1.73 (1.19-2.53)*	1.67 (1.08-2.59)*	1.12 (0.91-1.37)	1.50 (1.17-1.92)*	1.05 (0.91-1.22)	1.19 (0.99-1.42)	1.17 (1.08-1.27)*	1.29 (1.16-1.43)*
West	2.17 (1.62-2.90)*	2.23 (1.62-3.07)*	1.08 (0.85-1.38)	1.12 (0.89-1.43)	2.04 (1.79-2.34)*	2.00 (1.75-2.29)*	0.85 (0.77-0.93)*	0.88 (0.80-0.97)*
South	2.74 (2.12-3.55)*	2.25 (1.70-2.96)*	1.70 (1.43-2.03)*	1.58 (1.30-1.92)*	2.70 (2.43-3.00)*	2.13 (1.89-2.41)*	1.75 (1.63-1.88)*	1.47 (1.36-1.59)*
**Milk or curd (ref: daily)**								
Weekly	0.87 (0.67-1.12)	0.98 (0.74-1.28)	0.88 (0.73-1.06)	0.99 (0.81-1.21)	0.83 (0.76-0.90)*	0.96 (0.88-1.05)	0.83 (0.78-0.87)*	0.97 (0.92-1.03)
Never/occasionally	0.84 (0.65-1.08)	1.02 (0.76-1.36)	0.86 (0.72-1.01)	1.00 (0.82-1.23)	0.86 (0.78-0.94)*	1.04 (0.93-1.15)	0.75 (0.71-0.79)*	0.89 (0.84-0.94)*
**Pulses/beans (ref: daily)**								
Weekly	0.85 (0.69-1.04)	0.77 (0.60-0.98)*	0.90 (0.78-1.04)	0.87 (0.74-1.02)	1.07 (0.99-1.15)	1.01 (0.93-1.09)	0.89 (0.85-0.93)*	0.93 (0.88-0.97)*
Never/occasionally	0.80 (0.57-1.13)	0.74 (0.46-1.18)	0.96 (0.75-1.24)	1.12 (0.83-1.51)	0.95 (0.83-1.09)	0.93 (0.80-1.07)	0.99 (0.92-1.08)	1.12 (1.02-1.22)*
**Green leafy vegetables (ref: daily)**								
Weekly	1.12 (0.92-1.36)	1.25 (0.99-1.57)	1.12 (0.96-1.30)	1.28 (1.08-1.52)*	1.21 (1.12-1.30)*	1.07 (0.98-1.16)	1.02 (0.97-1.06)	0.98 (0.94-1.04)
Never/occasionally	1.26 (0.77-2.05)	1.52 (0.85-2.73)	0.91 (0.73-1.15)	1.03 (0.80-1.34)	1.28 (1.13-1.45)*	1.19 (1.04-1.36)*	1.01 (0.94-1.09)	0.98 (0.90-1.06)
**Fruits (ref: daily)**								
Weekly	1.04 (0.72-1.49)	1.01 (0.68-1.48)	0.82 (0.67-1.01)	0.98 (0.79-1.21)	0.98 (0.87-1.10)	1.02 (0.90-1.15)	0.77 (0.72-0.82)*	0.96 (0.90-1.02)
Never/occasionally	0.85 (0.61-1.20)	0.89 (0.61-1.29)	0.65 (0.53-0.80)*	0.89 (0.71-1.10)	0.87 (0.78-0.97)*	0.95 (0.84-1.08)	0.63 (0.59-0.67)*	0.92 (0.86-0.99)*
**Eggs (ref: daily)**								
Weekly	1.23 (0.84-1.80)	1.24 (0.83-1.86)	0.63 (0.50-0.79)*	0.68 (0.53-0.87)*	0.94 (0.79-1.11)	0.99 (0.84-1.16)	0.75 (0.69-0.82)*	0.89 (0.81-0.97)*
Never/occasionally	1.13 (0.76-1.67)	1.30 (0.85-1.99)	0.64 (0.51-0.80)*	0.82 (0.63-1.06)	0.70 (0.59-0.82)*	0.91 (0.76-1.09)	0.61 (0.56-0.67)*	0.88 (0.80-0.98)*
**Fish (ref: daily)**								
Weekly	0.81 (0.53-1.25)		0.90 (0.70-1.16)		1.08 (0.89-1.31)	1.25 (1.04-1.51)*	0.77 (0.70-0.84)*	1.05 (0.95-1.17)
Never/occasionally	0.85 (0.56-1.28)		0.88 (0.68-1.13)		0.86 (0.71-1.03)	1.29 (1.07-1.55)*	0.57 (0.53-0.63)*	0.91 (0.82-1.01)
**Chicken/meat (ref: daily)**								
Weekly	1.41 (0.71-2.83)		0.81 (0.55-1.19)		0.87 (0.61-1.25)	0.75 (0.53-1.06)	0.89 (0.74-1.06)	0.92 (0.75-1.13)
Never/occasionally	1.32 (0.66-2.65)		0.83 (0.56-1.21)		0.64 (0.45-0.91)*	0.67 (0.47-0.96)*	0.72 (0.60-0.85)*	0.96 (0.78-1.18)
**Fried food (ref: daily)**								
Weekly	0.92 (0.61-1.40)		1.02 (0.83-1.27)		1.14 (0.99-1.31)	1.11 (0.96-1.30)	0.81 (0.75-0.87)*	0.92 (0.84-1.00)
Never/occasionally	0.96 (0.66-1.38)		1.01 (0.83-1.24)		1.25 (1.09-1.43)*	1.15 (0.99-1.33)	0.79 (0.73-0.86)*	0.92 (0.84-1.01)
**Aerated drinks (ref: daily)**								
Weekly	1.24 (0.75-2.05)		0.85 (0.62-1.18)		0.96 (0.74-1.24)	0.86 (0.66-1.12)	1.02 (0.90-1.16)	0.99 (0.87-1.14)
Never/occasionally	1.26 (0.81-1.96)		0.87 (0.65-1.15)		1.10 (0.86-1.40)	0.99 (0.78-1.28)	0.92 (0.82-1.04)	0.96 (0.84-1.10)
**Tobacco usage (ref: no)**								
Yes	1.12 (0.89-1.40)		1.02 (0.87-1.20)		1.43 (1.19-1.71)*	1.23 (1.02-1.48)*	0.99 (0.89-1.11)	0.95 (0.85-1.06)
**Alcohol usage (ref: no)**								
Yes	1.51 (1.18-1.92)*	1.18 (0.92-1.53)	1.40 (1.21-1.63)*	1.03 (0.88-1.21)	1.03 (0.79-1.35)		0.90 (0.74-1.09)	
**Body mass index (ref: normal)**								
Underweight	0.69 (0.49-0.96)*	0.87 (0.62-1.23)	0.76 (0.60-0.97)*	1.01 (0.79-1.30)	0.67 (0.59-0.75)*	0.80 (0.71-0.90)*	0.63 (0.59-0.68)*	0.83 (0.77-0.89)*
Overweight	1.84 (1.41-2.40)*	1.54 (1.18-2.02)*	1.69 (1.40-2.04)*	1.34 (1.10-1.64)*	1.73 (1.55-1.93)*	1.41 (1.26-1.57)*	1.63 (1.53-1.74)*	1.22 (1.15-1.30)*
Obese1	2.41 (1.83-3.18)*	1.85 (1.41-2.43)*	2.28 (1.93-2.69)*	1.51 (1.26-1.81)*	2.89 (2.65-3.16)*	2.13 (1.94-2.34)*	2.44 (2.32-2.57)*	1.52 (1.44-1.61)*
Obese2	6.64 (4.57-9.67)*	5.08 (3.46-7.46)*	3.59 (2.64-4.90)*	2.15 (1.57-2.93)*	4.93 (4.46-5.45)*	3.38 (3.04-3.76)*	4.17 (3.92-4.45)*	2.05 (1.91-2.20)*
**Number of comorbidities (ref: more than one)**								
None	0.37 (0.17-0.82)*	0.54 (0.23-1.23)	0.08 (0.06-0.11)*	0.10 (0.07-0.15)*	0.48 (0.38-0.62)*	0.88 (0.69-1.13)	0.09 (0.08-0.10)*	0.15 (0.14-0.17)*
One	0.51 (0.21-1.24)	0.46 (0.18-1.15)	0.37 (0.25-0.54)*	0.33 (0.21-0.50)*	0.84 (0.65-1.09)	0.94 (0.73-1.22)	0.39 (0.35-0.43)*	0.43 (0.39-0.48)*
**Healthcare access (ref: yes)**								
No	0.85 (0.70-1.02)	1.05 (0.86-1.28)	0.68 (0.60-0.78)*	0.82 (0.71-0.94)*	0.90 (0.84-0.96)*	1.04 (0.96-1.11)	0.81 (0.78-0.85)*	1.01 (0.96-1.05)

RRR – relative risk ratio, CI – confidence interval

*Significant at *P* < 0.05

#### Females

The results indicate that the risk of undiagnosed diabetes and diagnosed diabetes increases with age in females. Individuals aged 45-49, have an adjusted RRR of 4.75 (95% CI = 4.11 to 5.50) for undiagnosed diabetes and 4.50 (95% CI = 4.15 to 4.87) for diagnosed diabetes compared to a younger age group (15-24), indicating an almost 5-fold higher risk. Higher wealth increased the risk of diagnosed diabetes, with those in the highest wealth index group having a 32% higher risk (adjusted RRR = 1.32; 95% CI = 1.20 to 1.46) than those in the lowest wealth index group. Women with no education have a 39% higher risk of undiagnosed diabetes compared to those with higher education (adjusted RRR = 1.39; 95% CI = 1.16 to 1.67). Women residing in urban areas have 12% higher risk of diagnosed diabetes compared to those in rural areas (adjusted RRR = 1.12; 95% CI = 1.05 to 1.19). Compared to those residing in the geographic north, females residing in the southern region had an almost two times higher risk of undiagnosed diabetes (adjusted RRR = 2.13; 95% CI = 1.89 to 2.41) and diagnosed diabetes (adjusted RRR = 1.47; 95% CI = 1.36 to 1.59).

Among modifiable risk factors, females who consume milk/curd never or occasionally have a 11% lower risk of diagnosed diabetes compared to daily consumers (adjusted RRR = 0.89; 95% CI = 0.85 to 0.93). Conversely, females who consume pulses/beans never/occasionally had 12% higher risk of diagnosed diabetes compared to daily consumers (adjusted RRR = 1.12; 95% CI = 1.02 to 1.22). Additionally, females who consume green leafy vegetables never/occasionally have a 19% higher risk of undiagnosed diabetes compared to daily consumers (adjusted RRR = 1.19; 95% CI = 1.04 to 1.36). Eating fruits never or occasionally lowers the risk of diagnosed diabetes by 8% compared to daily consumption (adjusted RRR = 0.92; 95% CI = 0.86 to 0.99). Females who consume eggs never or occasionally have a 12% lower risk of diagnosed diabetes by compared to daily consumers (adjusted RRR = 0.88; 95% CI = 0.80 to 0.98). Eating fish never or occasionally increases the risk of undiagnosed diabetes by 29% compared to daily consumption (adjusted RRR = 1.29; 95% CI = 1.07 to 1.55). Eating chicken/meat never or occasionally decreases the risk of undiagnosed diabetes by 33% compared to daily consumers (adjusted RRR = 0.67; 95% CI = 0.47 to 0.96).

Furthermore, female tobacco users have a 23% higher risk of undiagnosed diabetes than non-users (adjusted RRR = 1.23; 95% CI = 1.02 to 1.48). The risk of undiagnosed and diagnosed diabetes systematically increases with the BMI categories, with females in the obese 2 categories having the highest risk for both undiagnosed (adjusted RRR = 3.38; 95% CI = 3.04 to 3.76) and diagnosed diabetes (adjusted RRR = 2.05; 95% CI = 1.91 to 2.20). Moreover, females with no comorbidities had an 85% lower risk (adjusted RRR = 0.15; 95% CI = 0.14 to 0.17), and those with one comorbidity had a 57% lower risk (adjusted RRR = 0.43; 95% CI = 0.39 to 0.48) of diagnosed diabetes compared to females with more than one comorbidity.

#### Males

The results show that as age increases, the risk of undiagnosed diabetes and diagnosed diabetes increases for males. Males aged 45-49 have a 5-fold higher risk of both, undiagnosed diabetes (adjusted RRR = 5.24; 95% CI = 3.77 to 7.30) and diagnosed diabetes (adjusted RRR = 4.99; 95% CI = 3.96 to 6.28), compared to a younger age group 15-24. Males residing in the geographical south and west had a higher risk of undiagnosed diabetes (adjusted RRR = 2.25; 95% CI = 1.70 to 2.96) and (adjusted RRR = 2.23; 95% CI = 1.62 to 3.07), respectively compared to those in the north, while those in the east had a higher risk of diagnosed diabetes (adjusted RRR = 1.97; 95% CI = 1.47 to 2.59). Males in the highest wealth index group had a higher risk of diagnosed diabetes compared to the poorest wealth index group (adjusted RRR = 2.27; 95% CI = 1.67 to 3.09).

Among modifiable risk factors, males who consume pulses/beans weekly had a 23% lower risk of undiagnosed diabetes (adjusted RRR = 0.77; 95% CI = 0.60 to 0.98). Males who consume green leafy vegetables weekly had a 28% higher risk of diagnosed diabetes (adjusted RRR = 1.28; 95% CI = 1.08 to 1.52), but males who consume eggs weekly had a 32% lower risk of diagnosed diabetes (adjusted RRR = 0.68; 95% CI = 0.53 to 0.87). The risk of undiagnosed and diagnosed diabetes systematically increases with the BMI categories, with males in the obese 2 categories having the highest risk for both undiagnosed (adjusted RRR = 5.08; 95% CI = 3.46 to 7.46) and diagnosed diabetes (adjusted RRR = 2.15; 95% CI = 1.57 to 2.93). Males with no comorbidity (adjusted RRR = 0.10; 95% CI = 0.07 to 0.15) and one comorbidity (adjusted RRR = 0.33; 95% CI = 0.21 to 0.50) have 90% and 67% lower risk of diagnosed diabetes compared to males with more than one comorbidity. Males who do not have access to health care facilities have a 18% lower risk of diagnosed diabetes (adjusted RRR = 0.82; 95% CI = 0.71 to 0.94) compared to those with access to health care.

## DISCUSSION

The present study aimed to investigate the prevalence and risk factors associated with undiagnosed diabetes among a nationally representative sample of men and women aged 15-49 years in India. The study found that one in every four people with diabetes (24.82%) is not getting diagnosed, indicating a notable gap in identification and treatment of the disease. The findings revealed 4.90% prevalence of diabetes among the surveyed population. Interestingly, both undiagnosed diabetes and total diabetes were found to be equally prevalent among both genders, albeit with a higher prevalence observed in males. Middle-aged individuals (45-49 years), those in the lowest wealth index, those with higher BMI, those living in the geographical south of India were found to be at a higher risk of undiagnosed diabetes. Additionally, females with no education and tobacco users were also at a higher risk of undiagnosed diabetes. Certain dietary factors like lower intake of pulses/beans for males and lower intake of green leafy vegetables, fish and meat for females were found to be associated with a higher risk of undiagnosed diabetes.

The reported prevalence of diabetes from NFHS-5 survey based on our findings, is lower (4.9%) compared to estimates from other sources, such as the IDF 2019 (8.9%) [7], ICMR-INDIAB study (7.3%) [19] or the National Non-communicable Disease Monitoring Survey (NNMS) (9.3%) [10]. Also, notably, NFHS reports individuals with random blood glucose (RBS)>140 mg/dl, as having “high blood glucose” rather than as having diabetes [7-12]. However, it is important to note that we analysed the prevalence of undiagnosed and diagnosed diabetes among males and females below 50 years of age and non-pregnant females.

In the ICMR-INDIAB study, the reported prevalence of undiagnosed diabetes was as high as 47% among individuals with diabetes [19]. In our study, based on the recent NFHS-5 data, the overall prevalence of undiagnosed diabetes in India was similar to the NFHS-4 findings reported by Claypool et al. at 1.2% (95% CI = 1.2 to 1.3%) [11]. However, the proportion of undiagnosed diabetes has decreased from 42% as reported in NFHS-4 by Claypool et al. to approximately 25% in our study based on NFHS-5 data. The change in the proportion of undiagnosed cases between NFHS-4 and NFHS-5 surveys may result from various factors, including health care improvements, awareness, and population growth. It is important to note that Claypool et al. relied solely on self-reported diabetes to identify cases of undiagnosed diabetes, whereas our study employed a more comprehensive approach. In addition to self-reported diabetes, we incorporated two further criteria for defining diagnosed diabetes: a diagnosis confirmed by a health care professional and the use of medication for elevated blood glucose levels. This could have helped avoid potential overestimation of undiagnosed diabetes. Further, in the context of recent research on diabetes in India, Maiti et al. reported that among those with diabetes, only 27.5% (95% CI = 27.1 to 27.9%) were aware of their condition based on NFHS-5 data utilising a cut-off of >140 mg/dl to define diabetes [9]. Our study, in contrast to Maiti et al., employed a more robust definition of diabetes, which included opportunistic fasting glucose levels (≥126 mg/dl, with a diagnosed fasting duration of ≥8 hours) or random glucose levels (≥200 mg/dl).

In the context of previous studies on diabetes in India [2,3,7,10,12,20-22], which have identified that the risk of diabetes is higher among certain population subgroups, including rural residents, individuals with lower socioeconomic status, and those with lower levels of education, our findings further contribute to this understanding. We observed a higher risk of undiagnosed diabetes among specific subgroups, particularly rural females and females with lower levels of education. Additionally, we found that individuals in higher age groups, in the lower wealth index and those with a higher BMI, faced an increased risk of undiagnosed diabetes.

Prior research has highlighted obesity as a significant metabolic morbidity in India, and has demonstrated that the adherence to a prescribed diet plan and medications are indicative of glycaemic control as recommended by clinical guidelines [23]. A recent study has recommended that special attention be given to “rice-meat-refined wheat” dietary pattern due to its potential impact on central obesity and Hb1Ac in Indian populations, who are at a higher risk for cardiometabolic conditions compared to other populations [24]. Our study identified specific associations between dietary factors, BMI and undiagnosed diabetes risk among Indian males and females. Lower intake of pulses/beans for males and lower intake of green leafy vegetables, fish and meat for females were found to be associated with a higher risk of undiagnosed diabetes. Our study finding aligns with previous studies and shows that the risk of undiagnosed and diagnosed diabetes systematically increases with BMI, with those in the obese 2 categories having the highest risk for both undiagnosed and diagnosed diabetes.

Our findings underline the need for targeted diabetes prevention and control efforts to address the disparities in diabetes burden among different population subgroups in India. The study results provide important insights into the burden of undiagnosed diabetes in India, highlighting the need for interventions to improve diabetes screening, early detection of diabetes and access to health care to reduce the proportion of undiagnosed cases. The study also highlights the importance of addressing lifestyle and dietary factors in preventing and managing diabetes.

It is important to note that the study has several limitations, such as the fact that defining diabetes based on a single random blood glucose level using capillary blood glucose (CBG) and opportunistic fasting information may not meet the standards of diabetes diagnosis [25]. Additionally, the study did not include individuals above the age of 50, who are at a higher risk of developing diabetes, hence limits the generalisability of the study findings to older populations and comparisons with previous studies. Since, we only had access to random blood glucose readings, we could not distinguish between type 1 and type 2 diabetes. The estimates of undiagnosed diabetes are likely underestimated, and the extent of underestimation may vary based on the overrepresentation of random glucose measurements. Also, it is worth noting that the use of random blood glucose levels >200 mg/dl is generally reserved for symptomatic individuals and is advised to be confirmed by OGTT to determine their diabetes status [17]. Unfortunately, we were unable to confirm the diabetes status of these individuals in our study. Finally, the data set analysed in our study had a higher proportion of females and younger individuals, which may result in an underestimation or misrepresentation of the problem in these subgroups.

In a usual cross-sectional survey, the prevalence ratio (PR) is the measure of association used, and the RRR is often restricted as it may not accurately reflect the true association between the exposure and the outcome, as it would not take into account the potential for reverse causality or confounding. However, in this current study, to estimate the strength of the association between the exposure and the outcome, we have reported PR and RRR adjusted for confounders to control for factors that may bias the estimate of the association. The strength of the current study is that, in contrast to studies that have concentrated on specific states and cities, we underline that our research is typical and representative of all twenty-nine states and seven union territories in India and includes both urban and rural settings, as well as subgroups of states and union territories [20-22,26]. Further, we have used a more comprehensive approach to define diabetes and undiagnosed diabetes.

## CONCLUSION

The study provides important insights into the burden of undiagnosed diabetes in India, highlighting the need for interventions to improve diabetes screening and access to health care, as well as addressing lifestyle and dietary factors in the prevention and management of diabetes. This study also underlines the need for targeted diabetes prevention and control efforts to address the disparities in diabetes burden among different population subgroups in India. However, further research is needed to confirm the findings and explore the issue more comprehensively.

## Additional material


Online Supplementary Document

## References

[1] American Diabetes Association Improving care and promoting health in populations: Standards of Medical Care in Diabetes—2020. Diabetes Care. 2020;43 Suppl_1:S7-13. 10.2337/dc20-S001 31862744 PMC11869376

[2] International Diabetes Federation. IDF Diabetes Atlas. Brussels, Belgium; 2021.

[3] Anjana RM Pradeepa R Deepa M Datta M Sudha V Unnikrishnan R Prevalence of diabetes and prediabetes (impaired fasting glucose and/or impaired glucose tolerance) in urban and rural India: Phase I results of the Indian Council of Medical Research–INdia DIABetes (ICMR–INDIAB) study. Diabetologia. 2011;54:3022-7. 10.1007/s00125-011-2291-5 21959957

[4] Ramachandran A Snehalatha C Mary S Mukesh B Bhaskar AD Vijay V The Indian Diabetes Prevention Programme shows that lifestyle modification and metformin prevent type 2 diabetes in Asian Indian subjects with impaired glucose tolerance (IDPP-1). Diabetologia. 2006;49:289-97. 10.1007/s00125-005-0097-z 16391903

[5] Hills AP Arena R Khunti K Yajnik CS Jayawardena R Henry CJ Epidemiology and determinants of type 2 diabetes in South Asia. Lancet Diabetes Endocrinol. 2018;6:966-78. 10.1016/S2213-8587(18)30204-3 30287102

[6] Unnikrishnan R Anjana RM Mohan V Diabetes mellitus and its complications in India. Nat Rev Endocrinol. 2016;12:357-70. 10.1038/nrendo.2016.53 27080137

[7] International Institute for Population Sciences (IIPS). ICF. National Family Health Survey (NFHS-5), 2019-21: India. Mumbai; 2021.

[8] Joshi SR Diabetes Care in India. Ann Glob Health. 2015;81:830-8. 10.1016/j.aogh.2016.01.002 27108150

[9] Maiti S Akhtar S Upadhyay AK Mohanty SK Socioeconomic inequality in awareness, treatment, and control of diabetes among adults in India: Evidence from National Family Health Survey of India (NFHS), 2019–2021. Sci Rep. 2023;13:2971. 10.1038/s41598-023-29978-y 36805018 PMC9941485

[10] Mathur P Leburu S Kulothungan V Prevalence, Awareness, Treatment and Control of Diabetes in India From the Countrywide National NCD Monitoring Survey. Front Public Health. 2022;10:748157. 10.3389/fpubh.2022.748157 35359772 PMC8964146

[11] Claypool KT Chung M-K Deonarine A Gregg EW Patel CJ Characteristics of undiagnosed diabetes in men and women under the age of 50 years in the Indian subcontinent: the National Family Health Survey (NFHS-4)/Demographic Health Survey 2015–2016. BMJ Open Diabetes Res Care. 2020;8:e000965. 10.1136/bmjdrc-2019-000965 32098896 PMC7206915

[12] International Institute for Population Sciences (IIPS). ICF. National Family Health Survey (NFHS-4), 2015–16: India. Mumbai; 2017.

[13] Yesudian CA Grepstad M Visintin E Ferrario A The economic burden of diabetes in India: a review of the literature. Global Health. 2014;10:80. 10.1186/s12992-014-0080-x 25443136 PMC4279984

[14] WHO Expert Consultation Appropriate body-mass index for Asian populations and its implications for policy and intervention strategies. Lancet. 2004;363:157-63. 10.1016/S0140-6736(03)15268-3 14726171

[15] Bajaj S RSSDI clinical practice recommendations for the management of type 2 diabetes mellitus 2017. Int J Diabetes Dev Ctries. 2018;38:1-115. 10.1007/s13410-018-0604-7 29527102 PMC5838201

[16] Resnick HE Harris MI Brock DB Harris TB American Diabetes Association diabetes diagnostic criteria, advancing age, and cardiovascular disease risk profiles: results from the Third National Health and Nutrition Examination Survey. Diabetes Care. 2000;23:176-80. 10.2337/diacare.23.2.176 10868827

[17] Tandon N, Mohan V. ICMR guidelines for management of type 2 diabetes. 2018. 2022.

[18] StataCorp. Stata Statistical Software: Release 17. College Station, TX: StataCorp LLC; 2019.

[19] Anjana RM Deepa M Pradeepa R Mahanta J Narain K Das HK Prevalence of diabetes and prediabetes in 15 states of India: results from the ICMR-INDIAB population-based cross-sectional study. Lancet Diabetes Endocrinol. 2017;5:585-96. 10.1016/S2213-8587(17)30174-2 28601585

[20] Kutty VR Soman CR Joseph A Pisharody R Vijayakumar K Type 2 diabetes in southern Kerala: variation in prevalence among geographic divisions within a region. Natl Med J India. 2000;13:287-92. 11209482

[21] Tripathy JP Thakur J Jeet G Chawla S Jain S Pal A Prevalence and risk factors of diabetes in a large community-based study in North India: results from a STEPS survey in Punjab, India. Diabetol Metab Syndr. 2017;9:8. 10.1186/s13098-017-0207-3 28127405 PMC5259959

[22] Zargar AH Khan AK Masoodi SR Laway BA Wani AI Bashir MI Prevalence of type 2 diabetes mellitus and impaired glucose tolerance in the Kashmir Valley of the Indian subcontinent. Diabetes Res Clin Pract. 2000;47:135-46. 10.1016/S0168-8227(99)00110-2 10670914

[23] Masood MQ Singh K Kondal D Ali MK Mawani M Devarajan R Factors affecting achievement of glycemic targets among type 2 diabetes patients in South Asia: Analysis of the CARRS trial. Diabetes Res Clin Pract. 2021;171:108555. 10.1016/j.diabres.2020.108555 33242515 PMC7854496

[24] Cao Y Huynh Q Kapoor N Jeemon P Mello GT Oldenburg B Associations between Dietary Patterns and Cardiometabolic Risk Factors—A Longitudinal Analysis among High-Risk Individuals for Diabetes in Kerala, India. Nutrients. 2022;14:662. 10.3390/nu14030662 35277021 PMC8838960

[25] American Diabetes Association Classification and diagnosis of diabetes: Standards of medical care in diabetes—2018. Diabetes Care. 2018;41 Suppl_1:S13-27. 10.2337/dc18-S002 29222373

[26] Geldsetzer P Manne-Goehler J Theilmann M Davies JI Awasthi A Vollmer S Diabetes and hypertension in India: a nationally representative study of 1.3 million adults. JAMA Intern Med. 2018;178:363-72. 10.1001/jamainternmed.2017.8094 29379964 PMC5885928

