# Supplementary material for: Prevalence and risk factors associated with undiagnosed diabetes in India: Insights from NFHS-5 national survey
Source: J Glob Health. 2023 Dec 8;13:04135. doi: 10.7189/jogh.13.04135 (PMC10704946; doi:10.7189/jogh.13.04135)
Supplement: Online Supplementary Document [file jogh-13-04135-s001.pdf]

Of manuscript: Sahadevan, P., Kamal, V. K., Sasidharan, A., Bagepally, B. S., Kumari, D., & Pal, A. Prevalence and risk factors associated with undiagnosed diabetes in India: Insights from NFHS-5 national survey [Online supplementary document]

Contents

Figure S1: Flowchart of Cohort Selection from NFHS-5 (2019-2021)……………………….2

Table S1: General Characteristics of the Study population……………………………………3

Table S2: Undiagnosed diabetes prevalence among the states in India…………………..5

Table S3: Prevalence of undiagnosed diabetes in India by state for those with healthcare access……6

Table S4: Demographic Characteristics of Self-Reported and Undiagnosed Individuals by Gender with Chi-Square Test Results…………………………………………………………………7

Table S5: Proportion of Undiagnosed to Total Diabetes by Indian States………………10

Table S6: Association of Diabetes with other predictor variables stratified by gender………12

Appendix S1……….16

Appendix S2………………………………………………………………………………………………………..……16

Figure S1: Flowchart of Cohort Selection from NFHS-5 (2019-2021)

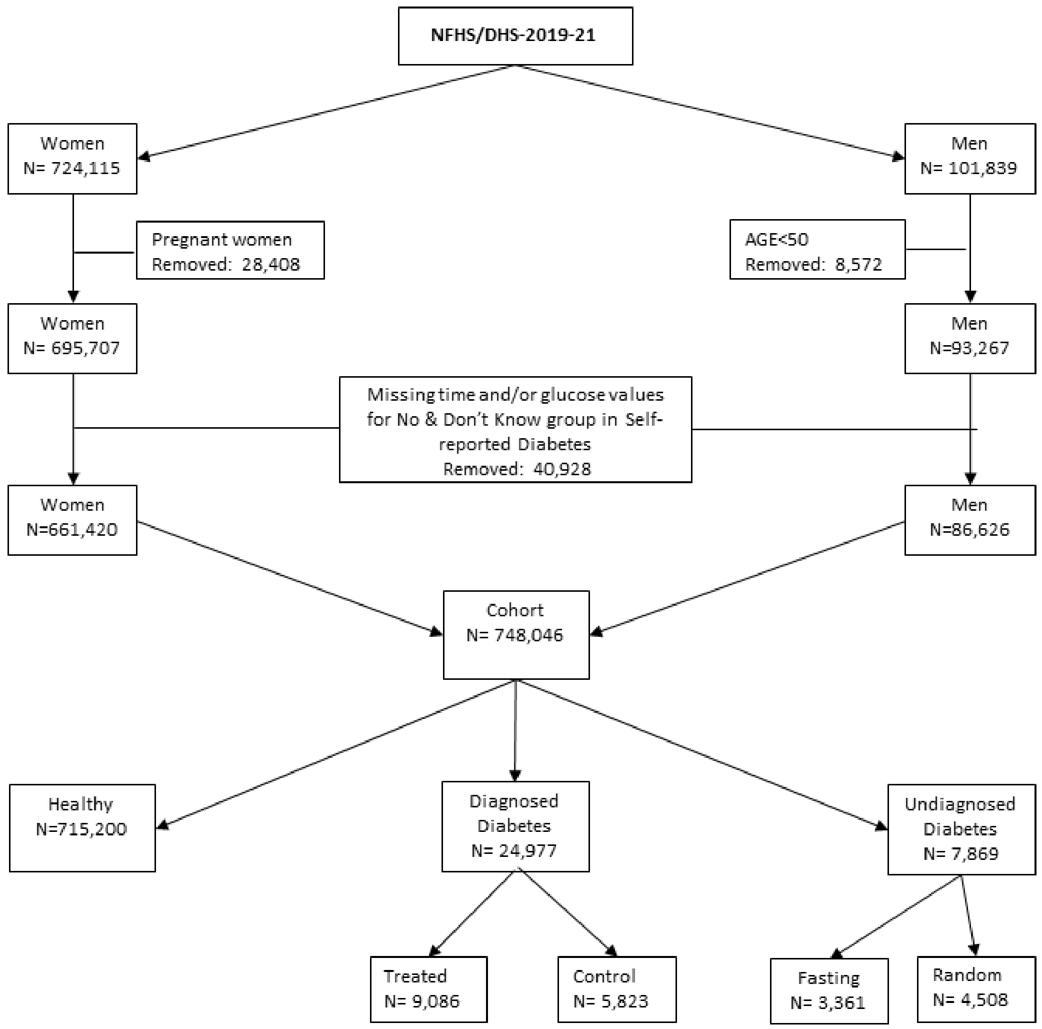

Table S1: General Characteristics of the Study Population

Characteristics	Male	Female	Overall
**Age in years: Mean (SD)**	30.43(9.93)	30.63(9.96)	30.61(9.95)
	**Male N (%)**	**Female N (%)**	**Overall N (%)**
**Age Categories**			
**15-24**	28,808(33.26)	214,803(32.48)	243,611(32.57)
**25-34**	25,666(29.63)	197,534(29.87)	223,200(29.84)
**35-44**	22,114(25.53)	169,404(25.61)	191,518(25.60)
**45-49**	10,038(11.59)	79,679(12.05)	89,717(11.09)
**Wealth index**			
**Poorest**	17,070(19.71)	137,009(20.71)	154,079(20.60)
**Poorer**	19,588(22.61)	147,524(22.30)	167,112(22.34)
**Middle**	18,623(21.50)	139,549(21.10)	158,172(21.14)

Richer	17,067(19.70)	127,781(19.32)	144,848(19.36)
Richest	14,278(16.48)	109,557(16.56)	123,835(16.55)
Education			
No education	9,303(10.74)	154,768(23.40)	164,071(21.93)
Primary	9,526(11.00)	78,340(11.84)	87,866(11.75)
Secondary	52,241(60.31)	337,523(51.03)	389,764(52.10)
Higher	15,556(17.96)	90,789(13.73)	106,345(14.22)
Place of residence			
Urban	21,915(25.30)	161,734(24.45)	183,649(24.55)
Rural	64,711(74.40)	499,686(75.55)	564,397(75.45)
Region of country			
North	17,957(20.73)	134,004(20.26)	151,961(20.31)
Central	19,657(22.69)	152,701(23.09)	172,358(23.04)
East	12,956(14.96)	108,307(16.37)	121,263(16.21)
Northeast	12,987(14.99)	95,620(14.46)	108,607(14.52)
West	9,994(11.54)	66,806(10.10)	76,800(10.27)
South	13,075(15.09)	103,982(15.72)	117,057(15.65)
Milk or curd			
Never/Occasionally	21,340(24.63)	202,265(30.58)	223,605(29.89)
Daily	39,651(45.77)	300,447(45.42)	340,098(45.46)
Weekly	25,635(29.59)	158,708(24.00)	184,343(24.64)
Pulses or beans			
Never/Occasionally	7,448(8.60)	58,541(8.85)	65,980(8.82)
Daily	40,170(46.37)	314,603(47.56)	354,773(47.43)
Weekly	39,008(45.03)	288,276(43.58)	327,284(43.75)
Green leafy vegetables			
Never/Occasionally	7,433(8.58)	62,096(9.39)	69,529(9.29)
Daily	44,292(51.13)	355,117(53.69)	399,409(53.39)
Weekly	34,901(40.29)	244,207(36.92)	279,108(37.31)
Fruits			
Never/Occasionally	40,706(46.99)	341,065(51.57)	381,771(51.04)
Daily	9,569(11.05)	75,375(11.40)	84,944(11.36)
Weekly	36,351(41.96)	244,980(37.04)	281,331(37.61)
Eggs			
Never/Occasionally	42,478(49.04)	378,412(57.21)	420,890(56.27)
Daily	5,117(5.91)	30,680(4.64)	35,797(4.79)
Weekly	39,031(45.06)	252,328(38.15)	291,359(38.95)
Fish			
Never/Occasionally	52,709(60.85)	442,151(66.85)	494,860(66.15)
Daily	3,817(4.41)	28,917(4.37)	32,734(4.38)
Weekly	30,100(34.75)	190,352(28.78)	220,452(29.47)
Chicken or meat			
Never/Occasionally	50,517(58.32)	431,439(65.23)	481,956(64.43)
Daily	1,842(2.13)	10,028(1.52)	11,870(1.59)
Weekly	34,267(39.56)	219,953(33.25)	254,220(33.98)
Fried food			
Never/Occasionally	48,691(56.21)	375,677(56.80)	424,368(56.73)

Daily	9,380(10.83)	62,630(9.47)	72,010(9.63)
Weekly	28,555(32.96)	223,113(33.73)	251,668(33.64)
Aerated drinks			
Never/Occasionally	66,749(77.05)	555,167(83.94)	621,916(83.14)
Daily	3,561(4.11)	20,322(3.07)	2,3883(3.19)
Weekly	16,316(18.83)	555,167(83.94)	621,916(83.14)
Tobacco usage			
No	56,515(65.24)	638,060(96.47)	694,575(92.85)
Yes	30,111(34.76)	23,360(3.53)	53,471(7.15)
Alcohol usage			
No	64,478(74.43)	648,769(98.09)	713,247(95.35)
Yes	22,148(25.57)	12,651(1.91)	34,799(4.65)
Body Mass Index			
Underweight (<18.5)	13,006(15.08)	120,053(18.20)	133,059(17.84)
Normal (18.50-22.99)	39,293(45.55)	299,512(45.42)	338,805(45.43)
Overweight (23-24.99)	15,361(17.81)	95,557(14.49)	110,918(14.87)
Obese1 (25-29.99)	15,639(18.13)	109,170(16.55)	124,809(16.74)
Obese2 (≥30)	2,972(3.44)	35,198(5.34)	38,170(5.12)
Comorbidities			
None	82,782(95.56)	606,044(91.63)	688,826(92.08)
One	3,353(3.87)	47,888(91.63)	688,826(92.08)
More than one	491(0.57)	7,488(1.13)	7,979(1.07)
Access to healthcare			
No	40,264(46.48)	353,456(53.44)	393,720(52.63)
Yes	46,362(53.52)	307,964(46.56)	354,326(47.37)

Table S2: Undiagnosed Diabetes Prevalence among Indian States

State/UT Name	MALE		FEMALE	
	PR	95% CI	PR	95% CI
**Andaman and Nicobar Islands**	0.0167	[.0066,.042]	0.0131	[.0075,.0228]
**Andhra Pradesh**	0.0269	[.0187,.0385]	0.0212	[.0184,.0244]
**Arunachal Pradesh**	0.0108	[.0065,.0179]	0.0053	[.0042,.0067]
**Assam**	0.0117	[.0077,.0177]	0.0066	[.0055,.0078]
**Bihar**	0.0118	[.0087,.0159]	0.0124	[.0110,.0140]
**Chandigarh**	0.0266	[.0061,.1078]	0.0189	[.0110,.0323]
**Chhattisgarh**	0.0168	[.0124,.0227]	0.0087	[.0073,.0104]
**Dadra and Nagar Haveli and Daman and Diu**	0.0490	[.0308,.0769]	0.0144	[.0092,.0225]
**Delhi**	0.0167	[.0101,.0275]	0.0085	[.0068,.0106]
**Goa**	0.0315	[.0163,.0599]	0.0060	[.0031,.0115]
**Gujarat**	0.0170	[.0130,.0222]	0.0112	[.0098,.0128]
**Haryana**	0.0111	[.0076,.0162]	0.0084	[.0070,.0101]
**Himachal Pradesh**	0.0112	[.0061,.0203]	0.0090	[.0068,.0119]
**Jammu and Kashmir**	0.00087	[0.0002, 0037]	0.0028	[.0020,.0040]
**Jharkhand**	0.0132	[.0092,.0189]	0.0093	[.0078,.0110]
**Karnataka**	0.0196	[.0142,.0270]	0.0107	[.0092,.0124]
**Kerala**	0.0124	[.0070,.0218]	0.0112	[.0091,.0138]
**Ladakh**				
**Lakshadweep**	0.0085	[.0012,.0576]	0.0087	[.0047,.0162]
**Madhya Pradesh**	0.0140	[.0109,.0179]	0.0085	[.0075,.0096]
**Maharashtra**	0.0185	[.0137,.0248]	0.0162	[.0142,.0184]
**Manipur**	0.0129	[.0067,.0246]	0.0115	[.0088,.015]
**Meghalaya**	0.0118	[.0057,.0246]	0.0026	[.0018,.0038]
**Mizoram**	0.0059	[.0027,.0129]	0.0046	[.0031,.0067]
**Nagaland**	0.0190	[.0107,.0337]	0.0156	[.012,.0203]
**Odisha**	0.0174	[.013,.0231]	0.0096	[.0083,.011]
**Puducherry**	0.0191	[.005,.0706]	0.0167	[.0112,.0249]
**Punjab**	0.0100	[.0065,.0152]	0.0086	[.0071,.0104]
**Rajasthan**	0.0075	[.0051,.011]	0.0058	[.0049,.0068]
**Sikkim**	0.0119	[.0029,.0480]	0.0031	[.0015,.0065]

**Tamil Nadu**	0.0280	[.0213,.0368]	0.0247	[.0224,.0272]
**Telangana**	0.0274	[.0209,.0359]	0.0220	[.0195,.0248]
**Tripura**	0.0432	[.0207,.0879]	0.0144	[.0112,.0186]
**Uttarakhand**	0.0108	[.0054,.0212]	0.0101	[.0075,.0135]
**Uttar Pradesh**	0.0095	[.0075,.0119]	0.0080	[.0072,.0088]
**West Bengal**	0.0131	[.0090,.0190]	0.0111	[.0094,.0132]

Table S3: Prevalence of undiagnosed diabetes in India by state for those with healthcare access

**State/UT Name**	**MALE**		**FEMALE**	
	**PR**	**95% CI**	**PR**	**95% CI**
**Andaman and Nicobar Islands**	0.0152	[.0020,.1067]	0.0122	[.0058,.0256]
**Andhra Pradesh**	0.0265	[.0186,.0376]	0.0213	[.0181,.0251]
**Arunachal Pradesh**	0.0147	[.0077,.0278]	0.0051	[.0035,.0073]
**Assam**	0.0095	[.0061,.0148]	0.0071	[.0058,.0087]
**Bihar**	0.0139	[.0088,.0219]	0.0122	[.0101,.0148]
**Chandigarh**	0.0264	[.0032,.1851]	0.0197	[.0075,.0508]
**Chhattisgarh**	0.0165	[.0114,.0238]	0.0097	[.0078,.0120]
**Dadra and Nagar Haveli and Daman and Diu**	0.0524	[.0286,.0943]	0.0164	[.0097,.0276]
**Delhi**	0.0177	[.0081,.0380]	0.0070	[.0048,.0102]
**Goa**	0.0343	[.0172,.0675]	0.0065	[.0031,.0135]
**Gujarat**	0.0159	[.0113,.0223]	0.0117	[.0096,.0142]
**Haryana**	0.0106	[.0057,.0197]	0.0083	[.0060,.0113]
**Himachal Pradesh**	0.0188	[.0090,.0391]	0.0087	[.0053,.0143]
**Jammu and Kashmir**			0.0023	[.0012,.0045]
**Jharkhand**	0.0161	[.0104,.0250]	0.0097	[.0079,.0119]
**Karnataka**	0.0189	[.0117,.0305]	0.0117	[.0094,.0146]
**Kerala**	0.0163	[.0089,.0295]	0.0114	[.0089,.0145]
**Ladakh**				
**Lakshadweep**	0.0146	[.0022,.0906]	0.0112	[.0061,.0207]
**Madhya Pradesh**	0.0158	[.0113,.0219]	0.0086	[.0071,.0103]

**Maharashtra**	0.0202	[.0127,.0319]	0.0173	[.0139,.0215]
**Manipur**	0.0130	[.0042,.0395]	0.0130	[.0083,.0203]
**Meghalaya**	0.0133	[.0056,.0310]	0.0033	[.0022,.0050]
**Mizoram**	0.0031	[0.00078, .0125]	0.0046	[.0031,.0068]
**Nagaland**	0.0090	[.0034,.0242]	0.0204	[.0126,.0327]
**Odisha**	0.0172	[.0120,.0247]	0.0102	[.0085,.0123]
**Puducherry**	0.0155	[.0022,.1018]	0.0168	[.0076,.0365]
**Punjab**	0.0103	[.0052,.0200]	0.0101	[.0073,.0139]
**Rajasthan**	0.0080	[.0054,.0118]	0.0060	[.0051,.0071]
**Sikkim**	0.0161	[.0019,.1215]	0.0052	[.0019,.0146]
**Tamil Nadu**	0.0258	[.0179,.0369]	0.0255	[.0224,.0291]
**Telangana**	0.0271	[.0209,.0350]	0.0241	[.0211,.0276]
**Tripura**	0.0342	[.0190,.0609]	0.0142	[.0105,.0191]
**Uttarakhand**	0.0113	[.0050,.0258]	0.0082	[.0059,.0115]
**Uttar Pradesh**	0.0095	[.0064,.0140]	0.0079	[.0066,.0093]
**West Bengal**	0.0119	[.0068,.0209]	0.0114	[.0091,.0142]

Table S4: Demographic Characteristics of Self-Reported and Undiagnosed Individuals by Gender with Chi-Square Test Results

	**MALE**				**FEMALE**			
**Characteristics**	**Self-reported N (%)**	**Undiagnosed N (%)**	**Total Diabetes N (%)**	***p* value**	**Self-reported N (%)**	**Undiagnosed N (%)**	**Total Diabetes N (%)**	***p* value**
**Age group**				<0.001				<0.001
**15-24**	425(14.16)	138(11.28)	563(13.33)		2922(13.30)	671(10.10)	3593(12.55)	
**25-34**	649(21.63)	272(22.24)	921(21.80)		4910(22.34)	1479(22.25)	6389(22.32)	
**35-44**	1108(36.92)	526(43.01)	1634(38.68)		8050(36.63)	2703(40.67)	10753(37.57)	
**45-49**	819(27.29)	287(23.47)	1106(26.18)		6094(27.73)	1793(26.98)	7887(27.56)	
**Wealth index**				0.001				<0.001
**Poorest**	362(12.06)	173(14.15)	535(12.67)		2925(13.31)	1115(16.78)	4040(14.12)	
**Poor**	539(17.96)	261(21.34)	800(18.94)		3922(17.85)	1309(19.70)	5231(18.28)	
**Middle**	662(22.06)	280(22.89)	942(22.30)		4574(20.81)	1501(22.59)	6075(21.22)	
**Richer**	735(24.49)	286(23.39)	1021(24.17)		5283(24.04)	1522(22.90)	6805(23.78)	

Richest	703(23.43)	223(18.23)	926(21.92)		5272(23.99)	1199(18.04)	6471(22.61)	
Education				0.004				<0.001
No education	311(10.36)	120(9.81)	431(10.20)		5485(24.96)	2165(32.58)	7650(26.73)	
Primary	319(10.63)	164(13.41)	483(11.43)		3037(13.82)	984(14.81)	4021(14.05)	
Secondary	1738(57.91)	729(59.61)	2467(58.40)		10553(48.02)	2912(43.82)	13465(47.04)	
Higher	633(21.09)	210(17.17)	843(19.96)		2901(13.20)	585(8.80)	3486(12.18)	
Place of residence				0.382				<0.001
Urban	974(32.46)	380(31.07)	1354(32.05)		7326(33.34)	1945(29.27)	9271(32.39)	
Rural	2027(67.54)	843(68.93)	2870(67.95)		14650(66.66)	4701(70.73)	19351(67.61)	
Region of country				<0.001				<0.001
North	578(19.26)	160(13.08)	738(17.47)		4635(21.09)	854(12.85)	5489(19.18)	
Central	525(17.49)	235(19.22)	760(17.99)		3991(18.16)	1226(18.45)	5217(18.23)	
East	519(17.29)	179(14.64)	698(16.52)		3446(15.68)	1117(16.81)	4563(15.94)	
Northeast	407(13.56)	159(13.00)	566(13.40)		3228(14.69)	708(10.65)	3936(13.75)	
West	278(9.26)	194(15.86)	472(11.17)		1699(7.73)	891(13.41)	2590(9.05)	
South	694(23.13)	296(24.20)	990(23.44)		4977(22.65)	1850(27.84)	6827(23.85)	
Milk or curd				0.776				<0.001
Never/Occasionally	676(22.53)	278(22.73)	954(22.59)		5611(25.53)	1891(28.45)	7502(26.21)	
Daily	1511(50.35)	602(49.22)	2113(50.02)		11366(51.72)	3321(49.97)	14687(51.31)	
Weekly	814(27.12)	343(28.05)	1157(27.39)		4999(22.75)	1434(21.58)	6433(22.48)	
Pulses or beans				0.069				<0.001
Never/Occasionally	227(7.56)	95(7.77)	322(7.62)		2034(9.26)	539(8.11)	2573(8.99)	
Daily	1546(51.52)	583(47.67)	2129(50.40)		10885(49.53)	3127(47.05)	14012(48.96)	
Weekly	1228(40.92)	545(44.56)	1773(41.97)		9057(41.21)	2980(44.84)	12037(42.06)	
Green leafy vegetable				0.503				<0.001
Never/Occasionally	222(7.40)	90(7.36)	312(7.39)		2002(9.11)	698(10.50)	2700(9.43)	
Daily	1585(52.82)	623(50.94)	2208(52.27)		11748(53.46)	3261(49.07)	15009(52.44)	
Weekly	1194(39.79)	510(41.70)	1704(40.34)		8226(37.43)	2687(40.43)	10913(38.13)	
Fruits				<0.001		698(10.50)	2700(9.43)	<0.001
Never/Occasionally	1195(39.82)	553(45.22)	1748(41.38)		9768(44.45)	3357(50.51)	13125(45.86)	
Daily	492(16.39)	149(12.18)	641(15.18)		3455(15.72)	757(11.39)	4212(14.72)	

**Weekly**	1314(43.79)	521(42.60)	1835(43.44)		8753(39.83)	2532(38.10)	11285(39.43)	
**Eggs**				0.005				<0.001
**Never/Occasionally**	1355(45.15)	575(47.02)	1930(45.69)		11614(52.85)	3477(52.32)	15091(52.73)	
**Daily**	266(8.86)	72(5.89)	338(8.00)		1470(6.69)	348(5.24)	1818(6.35)	
**Weekly**	1380(45.98)	576(47.10)	1956(46.31)		8892(40.46)	2821(42.45)	11713(40.92)	
**Fish**				0.585				<0.001
**Never/Occasionally**	1726(57.51)	720(58.87)	2446(57.91)		13420(61.07)	4268(64.22)	17688(61.80)	
**Daily**	164(5.46)	59(4.82)	223(5.28)		1486(6.76)	301(4.53)	1787(6.24)	
**Weekly**	1111(37.02)	444(36.30)	1555(36.81)		7070(32.17)	2077(31.25)	9147(31.96)	
**Chicken or meat**				0.099				0.014
**Never/Occasionally**	1666(55.51)	684(55.93)	2350(55.63)		13634(62.04)	4034(60.70)	17668(61.73)	
**Daily**	92(3.07)	23(1.88)	115(2.72)		438(1.99)	110(1.66)	548(1.91)	
**Weekly**	1243(41.42)	516(42.19)	1759(41.64)		7904(35.97)	2502(37.65)	10406(36.36)	
**Fried food**				0.225				<0.001
**Never/Occasionally**	1645(54.82)	706(57.73)	2351(55.66)		12184(55.44)	4004(60.25)	16188(56.56)	
**Daily**	331(11.03)	126(10.30)	457(10.82)		2417(11.00)	500(7.52)	2917(10.19)	
**Weekly**	1025(34.16)	391(31.97)	1416(33.52)		7375(33.56)	2142(32.23)	9517(33.25)	
**Aerated drinks**				0.008				<0.001
**Never/Occasionally**	2236(74.51)	959(78.41)	3195(75.64)		18021(82.00)	5664(85.22)	23685(82.75)	
**Daily**	160(5.33)	43(3.52)	203(4.81)		743(3.38)	171(2.57)	914(3.19)	
**Weekly**	605(20.16)	221(18.07)	826(19.55)		3212(14.62)	811(12.20)	4023(14.06)	
**Tobacco usage**				0.128				0.011
**No**	1978(65.91)	776(63.45)	2754(65.20)		21153(96.26)	6351(95.56)	27504(96.09)	
**Yes**	1023(34.09)	447(36.55)	1470(34.80)		823(3.74)	295(4.44)	1118(3.91)	
**Alcohol usage**				0.065				0.325
**No**	2075(69.14)	810(66.23)	2885(68.30)		21560(98.11)	6532(98.28)	28092(98.15)	
**Yes**	926(30.86)	413(33.77)	1339(31.70)		416(1.89)	114(1.72)	530(1.85)	
**Body Mass Index**				0.012				<0.001
**Underweight (<18.5)**	232(7.92)	102(8.38)	334(8.05)		1921(8.88)	612(9.23)	2533(8.97)	
**Normal (18.50-22.99)**	985(33.61)	366(30.07)	1351(32.57)		7223(33.40)	1982(29.90)	9205(32.58)	
**Overweight (23-24.99)**	606(20.68)	237(19.47)	843(20.32)		3580(16.56)	1028(15.51)	4608(16.31)	

**Obese1 (25-29.99)**	859(29.31)	372(30.57)	1231(29.68)		5873(27.16)	1930(29.12)	7803(27.62)	
**Obese2 (≥30)**	249(8.50)	140(11.50)	389(9.38)		3027(14.00)	1076(16.23)	4103(14.52)	
**Number of comorbidities**				<0.001				<0.001
**None**	2432(81.04)	1142(93.38)	3574(84.61)		15324(69.73)	5764(86.73)	21088(73.68)	
**One**	426(14.20)	68(5.56)	497(11.70)		4993(22.72)	763(11.48)	5756(20.11)	
**More than one**	143(4.77)	13(1.06)	156(3.69)		1659(7.55)	119(1.79)	1778(6.21)	
**Access to healthcare**				0.004				0.157
**No**	1152(38.39)	528(43.17)	1680(39.77)		10949(49.82)	3377(5.81)	14326(50.05)	
**Yes**	1849(61.61)	695(56.83)	2544(60.23)		11027(50.18)	3269(49.19)	14296(49.95)	

Table S5: Proportion of Undiagnosed among Total Diabetes by Indian States

**State/UT Name**	**MALE**		**FEMALE**	
	**PR**	**95%CI**	**PR**	**95%CI**
**Andaman and Nicobar Islands**	0.3778	[.133,.7063]	0.2315	[.1391,.3598]
**Andhra Pradesh**	0.3229	[.2255,.4385]	0.3038	[.2678,.3423]
**Arunachal Pradesh**	0.1877	[.1003,.3237]	0.1104	[.0865,.1399]
**Assam**	0.2395	[.1653,.3337]	0.1436	[.1216,.1689]
**Bihar**	0.2592	[.1907,.3419]	0.2770	[.2482,.3077]
**Chandigarh**	0.4831	[.1281,.856]	0.2750	[.1614,.4278]
**Chhattisgarh**	0.3704	[.2823,.4681]	0.2778	[.2345,.3257]
**Dadra and Nagar Haveli and Daman and Diu**	0.7078	[.5035,.8526]	0.3479	[.2536,.4559]
**Delhi**	0.2022	[.1269,.3065]	0.1828	[.1470,.2251]
**Goa**	0.5857	[.3389,.7958]	0.0914	[.0474,.1691]
**Gujarat**	0.4061	[.3178,.5009]	0.3184	[.2824,.3566]
**Haryana**	0.2363	[.1677,.3220]	0.1724	[.1445,.2044]
**Himachal Pradesh**	0.2849	[.1744,.4289]	0.2177	[.1678,.2776]
**Jammu and Kashmir**	0.0239	[.0057,.0943]	0.0529	[.0374,.0743]
**Jharkhand**	0.2302	[.1545,.3287]	0.2392	[.2003,.2829]
**Karnataka**	0.3671	[.2736,.4718]	0.2313	[.2023,.2632]

**Kerala**	0.1442	[.0821,.2408]	0.1454	[.1192,.1763]
**Ladakh**				
**Lakshadweep**	0.3108	[.0214,.9028]	0.1613	[.0899,.2723]
**Madhya Pradesh**	0.3629	[.2841,.4498]	0.2985	[.2696,.3291]
**Maharashtra**	0.3201	[.2507,.3985]	0.3621	[.3239,.4021]
**Manipur**	0.3539	[.1866,.5668]	0.3174	[.2464,.3982]
**Meghalaya**	0.2929	[.1420,.5090]	0.0642	[.0440,.0928]
**Mizoram**	0.1939	[.0826,.3913]	0.1390	[.0937,.2015]
**Nagaland**	0.5400	[.3364,.7311]	0.4644	[.3710,.5603]
**Odisha**	0.2913	[.2264,.3659]	0.2149	[.1860,.2469]
**Puducherry**	0.1884	[.0570,.4715]	0.2106	[.1403,.3036]
**Punjab**	0.2664	[.1775,.3792]	0.1776	[.1479,.2117]
**Rajasthan**	0.2517	[.1854,.3321]	0.1974	[.1697,.2284]
**Sikkim**	0.1853	[.0527,.4818]	0.0630	[.0306,.1253]
**Tamil Nadu**	0.3173	[.2556,.3861]	0.2437	[.2195,.2697]
**Telangana**	0.3104	[.2378,.3937]	0.3653	[.3289,.4033]
**Tripura**	0.5361	[.3067,.7512]	0.2894	[.2302,.3567]
**Uttarakhand**	0.2596	[.1076,.5050]	0.2546	[.1970,.3222]
**Uttar Pradesh**	0.2284	[.1844,.2792]	0.1946	[.1774,.2132]
**West Bengal**	0.2219	[.1522,.3118]	0.2060	[.1771,.2383]

Table S6: Association of Diabetes with other predictor variables stratified by gender.

	MALE				FEMALE			
**Characteristics**	**Healthy N(%)**	**Undiagnosed diabetes) N(%)**	**Self-reported diabetes N(%)**	**X^2 *p* value**	**Healthy N(%)**	**Undiagnosed diabetes N(%)**	**Self-reported diabetes N(%)**	**X^2 *p* value**
**Age group**				<0.001				<0.001
**15-24**	28245(34.28)	138(11.28)	425(14.16)		211210(33.38)	671(10.10)	2922(13.30)	
**25-34**	24745(30.03)	272(22.24)	649(21.63)		191145(30.21)	1479(22.25)	4910(22.34)	
**35-44**	20480(24.85)	526(43.01)	1108(36.92)		158651(25.07)	2703(40.67)	8050(36.63)	
**45-49**	8932(10.84)	287(23.47)	819(27.29)		71792(11.35)	1793(26.98)	6094(27.73)	
**Wealth Index**				<0.001				<0.001
**Poorest**	16535(20.07)	173(14.15)	362(12.06)		132969(21.01)	1115(16.78)	2925(13.31)	
**Poorer**	18788(22.80)	261(21.34)	539(17.96)		142293(22.49)	1309(19.70)	3922(17.85)	
**Middle**	17681(21.46)	280(22.89)	662(22.06)		133474(21.09)	1501(2.59)	4574(20.81)	
**Richer**	16046(19.47)	286(23.39)	735(24.49)		120976(19.12)	1522(22.90)	5283(24.04)	
**Richest**	13352(16.20)	223(18.23)	703(23.43)		103086(16.29)	1199(18.04)	5272(23.99)	
**Education level**				<0.001				<0.001
**No education**	8872(10.77)	120(9.81)	311(10.36)		147118(23.25)	2165(32.58)	5485(24.96)	
**Primary**	9043(10.97)	164(13.41)	319(10.63)		74319(11.74)	984(14.81)	3037(13.82)	
**Secondary**	49774(60.40)	729(59.61)	1738(57.91)		324058(51.21)	2912(43.82)	10553(48.02)	
**Higher**	14713(17.86)	210(17.17)	633(21.09)		87303(13.80)	585(8.80)	2901(13.20)	
**Place of Residence**				<0.001				<0.001
**Urban**	20561(24.95)	380(31.07)	974(32.46)		152463(24.09)	1945(29.27)	7326(33.34)	
**Rural**	61841(75.05)	843(68.93)	2027(67.54)		480335(75.91)	4701(70.73)	14650(66.56)	
**Region of country**				<0.001				<0.001
**North**	17219(20.90)	160(13.08)	578(17.49)		128515(20.31)	854(12.85)	4635(21.09)	
**Central**	18897(22.93)	235(19.22)	525(17.49)		147484(23.31)	1226(18.45)	3991(18.16)	

**East**	12258(14.88)	179(14.64)	519(17.29)		103744(16.39)	1117(16.81)	3446(15.68)	
**Northeast**	12421(15.07)	159(13.00)	407(13.56)		91684(14.49)	708(10.65)	3228(14.69)	
**West**	9522(11.56)	194(15.86)	278(9.26)		64216(10.15)	891(13.41)	1699(7.73)	
**South**	12085(14.67)	296(24.20)	694(23.13)		97155(15.35)	1850(27.84)	4977(22.65)	
**Milk or curd**				<0.001				<0.001
**Daily**	37538(45.55)	602(49.22)	1511(50.35)		285760(45.16)	3321(49.97)	11366(51.72)	
**Weekly**	24478(29.71)	343(28.05)	814(27.12)		152275(24.06)	1434(21.58)	4999(22.75)	
**Never/Occasionally**	20386(24.74)	278(22.73)	676(22.53)		194763(30.78)	1891(28.45)	5611(25.53)	
**Pulses/beans**				<0.001				<0.001
**Daily**	38041(46.17)	583(47.67)	1546(51.52)		300591(47.50)	3127(47.05)	10885(49.53)	
**Weekly**	37235(45.19)	545(44.56)	1228(40.92)		276239(43.69)	2980(44.84)	9057(41.21)	
**Never/Occasionally**	7126(8.65)	95(7.77)	227(7.56)		55964(8.84)	539(8.11)	2034(9.26)	
**Green leafy vegetables**				0.042				<0.001
**Daily**	42084(51.07)	623(50.94)	1585(52.82)		340108(53.75)	3261(49.07)	11748(53.46)	
**Weekly**	33197(40.29)	510(41.70)	1194(39.79)		233294(36.87)	2687(40.43)	8226(37.43)	
**Never/Occasionally**	7121(8.64)	90(7.36)	222(7.40)		59396(9.39)	698(10.50)	2002(9.11)	
**Fruits**				<0.001				<0.001
**Daily**	8928(10.83)	149(12.18)	492(16.39)		71163(11.25)	757(11.39)	3455(15.72)	
**Weekly**	34516(41.89)	521(42.60)	1314(43.79)		233695(36.93)	2532(38.10)	8753(39.83)	
**Never/Occasionally**	38954(47.28)	553(45.22)	1195(39.82)		327940(51.82)	3357(50.51)	9768(44.45)	
**Eggs**				<0.001				<0.001
**Daily**	4779(5.80)	72(5.89)	266(8.86)		28862(4.56)	348(5.24)	1470(6.69)	
**Weekly**	37075(44.99)	576(47.10)	1380(45.98)		240615(38.02)	2821(42.45)	8892(40.46)	
**Never/Occasionally**	40548(49.21)	575(47.02)	1355(45.15)		363321(57.42)	3477(52.32)	11614(52.85)	
**Fish**				<0.001				<0.001
**Daily**	3594(4.36)	59(4.82)	164(5.46)		27130(4.29)	301(4.53)	1486(6.76)	
**Weekly**	28545(34.64)	444(36.30)	1111(37.02)		181205(28.64)	2077(31.25)	7070(32.17)	
**Never/Occasionally**	50263(61.00)	720(58.87)	1726(57.51)		424463(67.08)	4268(64.22)	13420(61.07)	

**Chicken/meat**				<0.001				<0.001
**Daily**	1727(2.10)	23(1.88)	92(3.07)		9480(1.50)	110(1.66)	438(1.99)	
**Weekly**	32508(39.45)	516(42.19)	1243(41.42)		209547(33.11)	2502(37.65)	7904(35.97)	
**Never/Occasionally**	48167(58.45)	684(55.93)	1666(55.51)		413771(65.39)	4034(60.70)	13634(62.04)	
**Fried food**				0.455				<0.001
**Daily**	8923(10.83)	126(10.30)	331(11.03)		59713(9.44)	500(7.52)	2417(11.00)	
**Weekly**	27139(32.93)	391(31.97)	1025(34.16)		213596(33.75)	2142(32.23)	7375(33.56)	
**Never/Occasionally**	46340(56.24)	706(57.73)	1645(54.82)		359489(56.81)	4004(60.25)	12184(55.44)	
**Aerated drinks**				0.001				<0.001
**Daily**	3358(4.08)	43(3.52)	160(5.33)		19408(3.07)	171(2.57)	743(3.38)	
**Weekly**	15490(18.80)	221(18.07)	605(20.16)		81907(12.94)	811(12.20)	3212(14.62)	
**Never/Occasionally**	63554(77.13)	959(78.41)	2236(74.51)		531482(83.99)	5664(85.22)	18021(82.00)	
**Tobacco usage**				0.313				<0.001
**No**	53761(65.24)	776(63.45)	1978(65.91)		610556(96.49)	6351(95.56)	21153(96.26)	
**Yes**	28641(34.76)	447(36.55)	1023(34.09)		22242(3.51)	295(4.44)	823(3.74)	
**Alcohol usage**				<0.001				0.484
**No**	61593(74.75)	810(66.23)	2075(69.14)		620677(98.08)	6532(98.28)	21560(98.11)	
**Yes**	20809(25.25)	413(33.77)	926(30.86)		12121(1.92)	114(1.72)	416(1.89)	
**Body Mass Index**				<0.001				<0.001
**Underweight (<18.5)**	12672(15.43)	102(8.38)	232(7.92)		117520(18.62)	612(9.23)	1921(8.88)	
**Normal (18.50-22.99)**	37942(46.20)	366(30.07)	985(33.61)		290307(45.99)	1982(29.90)	7223(33.40)	
**Overweight (23-24.99)**	14518(17.68)	237(19.47)	606(20.68)		90949(14.41)	1028(15.51)	3580(16.56)	
**Obese1 (25-29.99)**	14408(17.54)	372(30.57)	859(29.31)		31095(4.93)	1076(16.23)	5873(27.16)	
**Obese2 (≥30)**	2583(3.15)	140(11.50)	249(8.50)				3027(14.00)	
**Number of comorbidities**				<0.001				<0.001
**None**	79208(96.12)	1142(93.38)	2432(81.04)		584956(92.44)	5764(86.73)	15324(69.73)	
**One**	2859(3.47)	68(5.56)	426(14.20)		42132(6.66)	763(11.48)	4993(22.72)	
**More than one**	335(0.41)	13(1.06)	143(4.77)		5710(0.90)	119(1.79)	1659(7.55)	

**Access to healthcare**				<0.001				<0.001
**No**	38584(46.82)	528(43.17)	1152(38.39)		339130(53.59)	3377(50.81)	10949(49.82)	
**Yes**	43818(53.18)	695(56.83)	1849(61.61)		293668(46.41)	3269(49.19)	11027(50.18)	

Appendix S1

The fifth round of the National Family Health Survey (NFHS-5), a nationally representative household-based survey was conducted in India during 2019–21. The survey covered 707 districts in 28 states and 8 union territories, and interviewed a total of 2,843,917 individuals from 636,699 households. Of these, 2,078,315 were adults above 15 years of age.

The NFHS-5 used a stratified two-stage sampling method. In the first stage, within each district, The sampling process was carried out differently in rural and urban areas. Villages were used as primary sampling units (PSUs) in rural areas, and census enumeration blocks (CEBs) were used as PSUs in urban areas. The PSUs were selected using probability proportional to size (PPS). In the second stage, 22 households were randomly selected from each selected PSU in every selected rural and urban cluster using systematic random sampling. This was done after the complete mapping and household listing of the selected PSUs.

The detailed methodology followed by NFHS-5 can be found in the NFHS India Report.

Appendix S2

Study variables

Socio-demographic Characteristics: Potential socio-demographic risk factors for diabetes in India were evaluated, including variables such as sex, age group (categorized in 10-year bins), wealth index (classified as poorest, poor, middle, richer, and richest), level of education (categorized as none, middle, richer, and richest), drinking and tobacco usage habits, and place of residence (categorized as rural and urban). Indian states and union territories were grouped into six administrative regions (North, Northeast, Central, South, East and West) to ensure adequate sample size within each region (with Central being the reference region). Body mass index (BMI) was categorized according to the cut-off points proposed by the WHO and Asia-Pacific guidelines: underweight (<18.5 kg/m2), normal weight (18.5-22.9 kg/m2), overweight

at risk (23-24.9 kg/m2), obese1 (25-29.9 kg/m2) and obese2 (≥30 kg/m2). (1)

Diabetes definitions: Our definition of self-reported diabetes was based on participants answering "yes" to the question of whether they currently have diabetes. Diagnosed diabetes was determined by participants' answering "yes" to any of the following questions: whether they currently have diabetes, whether they currently take a prescribed medication to lower blood glucose, or whether they were told by a doctor or other health professional that they have high blood glucose on two or more occasions.

We defined participants with undiagnosed diabetes as those who answered "no" or "don't know" to the diagnosed diabetes variable defined, and subsequently exhibited fasting or random glucose levels. Specifically, this included individuals with opportunistic fasting—those who self-reported not having eaten for 8 or more hours prior to the test and had an opportunistic fasting glucose level ≥126mg/dL or a random glucose level ≥200mg/dL. These are in accordance with guidelines established by the Indian Council of Medical Research, International diabetes Federation, American Diabetes Association, World Health Organization, US Preventive Services Task Force and the Research Society for the Study of Diabetes in India (RSSDI) guidelines of 2017 (2-6), which recommend the use of HbA1c, fasting plasma glucose level, and oral glucose tolerance test to screen for diabetes.

Treated was defined by answering "yes" to the question: whether they currently take a prescribed medication to lower blood glucose. Control was defined by answering "yes" to the question: whether they currently take a prescribed medication to lower blood glucose, and subsequently exhibited fasting or random glucose levels.

The NFHS-5 study employed the Accu-Chek Performa glucometer and corresponding test strips to obtain random blood glucose measurements from finger-stick specimens of all participants. Participants with blood glucose levels ≥200 mg/dl were referred for additional medical evaluation. The study did not include fasting instructions for participants but recorded

the time of their last meal or beverage.

Comorbid conditions: The survey assessed the self-reported prevalence of various chronic medical conditions, including diabetes, hypertension, asthma, thyroid disorder, heart disease, cancer, and chronic kidney disorder, among the interviewed participants. Self-reported cases were determined by affirmative responses ("yes") to specific questions pertaining to each condition.

Healthcare access: The NFHS-5 survey participants were solicited for information on their health insurance coverage, the sources of healthcare, and frequency of contact with healthcare workers or healthcare professionals via a series of questions. Healthcare access was operationalized as a binary construct, wherein affirmative responses ("yes") were required for either of the following: having health insurance or seeing a healthcare provider within the past 12 months, or visiting a healthcare facility within the past three months.

Dietary Predictor variables: Frequency of consumption of selected foods were evaluated through inquiry, with participants being asked to self-report their consumption habits for items such as fish, milk or curd, pulses or beans, green leafy vegetables, other vegetables and fruits, eggs, chicken, or meat, using response options of daily, weekly, occasionally or never.

References

1. Consultation WE. Appropriate body-mass index for Asian populations and its implications for policy and intervention strategies. Lancet (London, England). 2004;363(9403):157-63.
2. Association AD. 2. Classification and diagnosis of diabetes: standards of medical care in diabetes—2018. Diabetes care. 2018;41(Supplement_1):S13-S27.
3. Association AD. 1. Improving care and promoting health in populations: Standards of Medical Care in Diabetes—2020. Diabetes care. 2020;43(Supplement_1):S7-S13.

4. Bajaj S. RSSDI clinical practice recommendations for the management of type 2 diabetes mellitus 2017. International journal of diabetes in developing countries. 2018;38(1):1-115.
5. International Diabetes Federation. IDF Diabetes Atlas. Brussels, Belgium; 2021.
6. Tandon N, Mohan V. ICMR guidelines for management of type 2 diabetes. 2018. 2022.
